# Her9/Hes4 is required for retinal photoreceptor development, maintenance, and survival

**DOI:** 10.1038/s41598-020-68172-2

**Published:** 2020-07-09

**Authors:** Cagney E. Coomer, Stephen G. Wilson, Kayla F. Titialii-Torres, Jessica D. Bills, Laura A. Krueger, Rebecca A. Petersen, Evelyn M. Turnbaugh, Eden L. Janesch, Ann C. Morris

**Affiliations:** Department of Biology, University of Kentucky, 215 T.H. Morgan Building, Lexington, KY 40506-0225 USA

**Keywords:** Differentiation, Zebrafish, Developmental neurogenesis

## Abstract

The intrinsic and extrinsic factors that regulate vertebrate photoreceptor specification and differentiation are complex, and our understanding of all the players is far from complete. Her9, the zebrafish ortholog of human HES4, is a basic helix-loop-helix-orange transcriptional repressor that regulates neurogenesis in several developmental contexts. We have previously shown that *her9* is upregulated during chronic rod photoreceptor degeneration and regeneration in adult zebrafish, but little is known about the role of *her9* during retinal development. To better understand the function of Her9 in the retina, we generated zebrafish *her9* CRISPR mutants. *Her9* homozygous mutants displayed striking retinal phenotypes, including decreased numbers of rods and red/green cones, whereas blue and UV cones were relatively unaffected. The reduction in rods and red/green cones correlated with defects in photoreceptor subtype lineage specification. The remaining rods and double cones displayed abnormal outer segments, and elevated levels of apoptosis. In addition to the photoreceptor defects, *her9* mutants also possessed a reduced proliferative ciliary marginal zone, and decreased and disorganized Müller glia. Mutation of *her9* was larval lethal, with no mutants surviving past 13 days post fertilization. Our results reveal a previously undescribed role for Her9/Hes4 in photoreceptor differentiation, maintenance, and survival.

## Introduction

The vertebrate retina is a highly conserved tissue of the central nervous system (CNS) that captures and converts light into an electrical signal. The neural retina is composed of three layers; the ganglion cell layer (GCL), inner nuclear layer (INL) and outer nuclear layer (ONL); the light-sensitive rod and cone photoreceptors reside in the ONL. The six classes of retinal neurons and single glial cell type in the retina all differentiate from a single pool of multipotent retinal progenitor cells (RPCs). Across all vertebrates studied to date, specification and differentiation of retinal cell types occurs in a synchronized and largely conserved manner. The ganglion cells are born first and the rods, bipolar cells, and Müller glia differentiate last^1^. Cone photoreceptor differentiation generally occurs before the rods. The development of photoreceptor cell identity is influenced by many factors, both intrinsic and extrinsic. Cone and rod photoreceptor specification, cell cycle exit, and differentiation are accompanied by the expression of transcription factors such as *otx2* and *crx*, then rod or cone-specific factors such as *Nr2e3* and *trβ2*^2,3^. Photoreceptor differentiation is also regulated extrinsically by Hedgehog (Hh), thyroid hormone (TH), Notch, Wnt, retinoic acid (RA), and fibroblast growth factor (FGF) signaling pathways, to name a few^4^. Our knowledge of the precise molecular determinants of photoreceptor subtype differentiation is far from complete. Understanding how photoreceptor cell development is regulated is essential for understanding the pathogenesis of blinding retinal degenerative diseases such as retinitis pigmentosa and macular degeneration. Several approaches have been developed to understand retinal development, one of the most prominent being the generation and characterization of genetic mutants. Due to its conserved retinal structure and function, as well as the extensive genetic homology with humans, zebrafish have become an appealing model for interrogating the role of specific genes in retinal cell type differentiation.

In this study, we examine the role of the transcription factor *Hairy and enhancer of split related 9* (Her9) in retinal development. Her genes belong to a family of basic-Helix-Loop-Helix-Orange (bHLH-O) DNA binding proteins^5^, and are homologs of the *Hairy* and *Enhancer-of-split* genes in Drosophila and of *Hes/Hey* genes in mammals. The zebrafish genome contains 19 *hairy-related (her)* genes which have been shown to play essential roles in developmental processes such as somitogenesis, neural tube and nervous system development, floor plate development, cell cycle exit, and apoptosis^6,7^. Her proteins often function as downstream effectors of the Notch-Delta signaling pathway and mediate cross-talk between Notch and other pathways^8^. A variety of members in the three *hairy* classes are also regulated by Hh, VEGF, Nodal, FGF, and RA signaling^9–13^.

Zebrafish Her9 shares homology with mouse and human Hes1^9^, but is more closely related by sequence comparison and synteny analysis to human HES4^14^. As Hes4 is absent from the mouse and rat genomes, zebrafish provide a unique opportunity to explore the function of this transcription factor during retinal development. In zebrafish, *her9* is expressed in ectodermal tissues^15^ and in inter-pro-neural domains during embryonic development^16^, where morpholino knockdown suggested it acts downstream of Bmp signaling. Recent studies have also shown that Her9 functions during inner ear development, where it defines the posterolateral non-neurogenic field downstream of *tbx1* expression and RA signaling^9^. Interestingly, *her9* expression was shown to be independent of Notch-Delta signaling in several developmental contexts^15,16^, suggesting that it does not function as a classical Notch effector gene. In the post-embryonic Medaka and Xenopus retina, Her9 regulates proliferation of retinal stem cells in the peripheral ciliary marginal zone (CMZ)^14,17^. Finally, *her9* expression is upregulated during the specific degeneration and regeneration of rod photoreceptors in the zebrafish retina, suggesting a role for this transcription factor in retinal regeneration^18^. Although these studies strongly implicate *her9* in regulating continual neurogenesis and injury-induced regeneration in the post-embryonic retina, the function of *her9* during embryonic retinal development has not been thoroughly investigated.

In this study, we generated *her9* mutants using the CRISPR/Cas9 system to determine whether *her9* has a required role in retinal development. Here, we demonstrate that loss of Her9 causes a significant decrease in rod photoreceptors, subsets of cone photoreceptors, Müller glia cells, and CMZ size. We also find that *her9* mutant photoreceptors have abnormal outer segments and undergo apoptosis. Finally, we show that *her9* expression in the retina is regulated by RA signaling, but RA responsiveness is not completely lost in *her9* mutants. Taken together, our study confirms the requirement for Her9/HES4 in retinal stem cell proliferation and identifies Her9/HES4 as a novel regulator of photoreceptor specification, maintenance, and survival.

## Results

### *Her9* expression during retinal development

**Figure 1:**
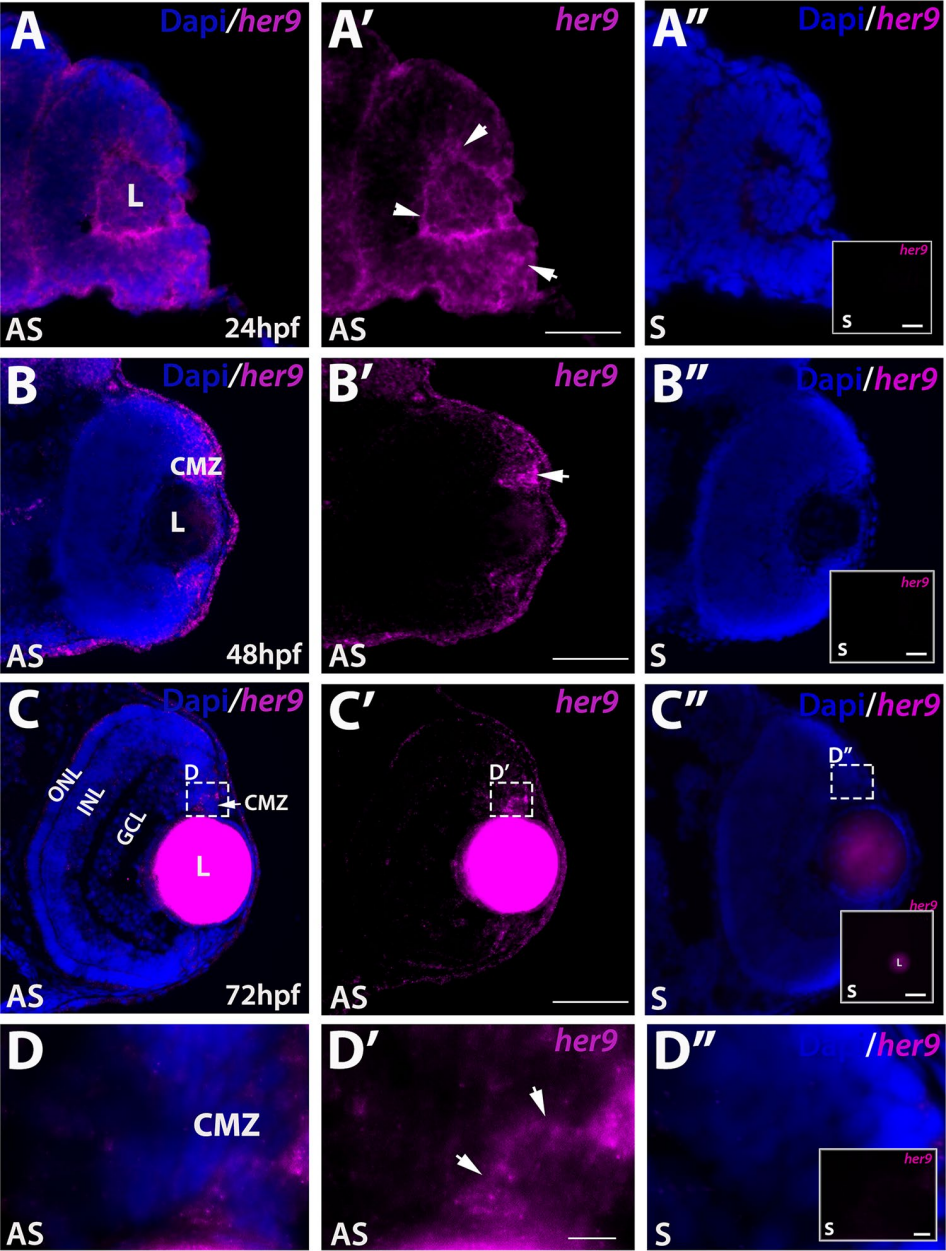
*Her9* expression in the developing retina. Fluorescent in situ hybridization (FISH) showing *her9* mRNA expression at 24 (**A**–**A′**), 48 (**B**–**B′**), and 72 (**C**–**C′**) hpf in the developing retina (expression in the lens at 72 hpf is auto-fluorescence). (**D**–**D′**) × 100 magnification of boxed area in (**C**–**C′**) showing *her9* expression in the CMZ. *her9* sense probes at 24, 48, and 72 hpf are shown in (**A″**, **B″**, **C″**, **D″**). Panels in (**A″**–**D″**) display *her9* sense probe without the DAPI staining. L, lens CMZ, ciliary marginal zone; NR, neural retina; ONL, outer nuclear layer; INL, inner nuclear layer; GCL, ganglion cell layer. Scale bar = 50 μm and 100 μm.

Previous studies in zebrafish have described *her9* expression patterns in the early embryo^10,15,16^ and *her9* expression has also been documented in the CMZ of the post-embryonic medaka retina^17^. We investigated the expression of *her9* during the critical stages of retina development. We used fluorescent in situ hybridization (FISH) to detect *her9* mRNA in the developing zebrafish retina between 24 and 72 hpf, a time period which encompasses the progression from a pseudostratified proliferative retinal neuro-epithelium to a fully laminated, functional retina containing all retinal cell types. At 24 hpf, *her9* was expressed throughout the lens and retina, but most predominately in the ventral portion of the retina, and in a ring around the lens (Fig. 1A–A″). At 48 hpf, *her9* expression became restricted to the undifferentiated peripheral retina (Fig. 1B–B″). By 72 hpf, *her9* expression was only detected in a small patch of cells within the persistently neurogenic CMZ (Fig. 1C–D″). Taken together, the expression of *her9* throughout the undifferentiated embryonic retina and in the proliferative CMZ suggests that it functions in retinal progenitor cells and potentially plays a role in retinal stem cell proliferation or maintenance.

### Generating *her9* mutants using CRISPR/Cas9

To explore the role of *her9* during retinal development, we used the CRISPR/Cas9 system to introduce mutations in the *her9* gene, targeting a region of exon 1 upstream of the bHLH domain (Fig. S1A). We recovered two *her9* mutant alleles, one carrying a 1-bp deletion and the other a 1-bp insertion at the target site (Fig. S1B). Both mutations cause a frameshift resulting in a premature stop codon. Both mutations introduced novel restriction sites, allowing for genotyping by RFLP analysis (Fig. S1C). We used qPCR to investigate *her9* expression in *her9*^*−/−*^ zebrafish, and observed a significant reduction in *her9* mRNA levels, suggesting that the mutations resulted in nonsense-mediated decay (Fig. S1D). We also used a HES4 antibody to detect Her9 protein, which confirmed a significant decrease in expression in *her9* mutants (Fig. S1E). These results indicate that the CRISPR-generated genetic lesion in *her9* is a loss of function mutation. Both mutant alleles presented similar phenotypes (Fig. S1F–G) so here we present data collected from the 1 bp insertion mutant.

### Characterization of the *her9* mutant phenotype

**Figure 2:**
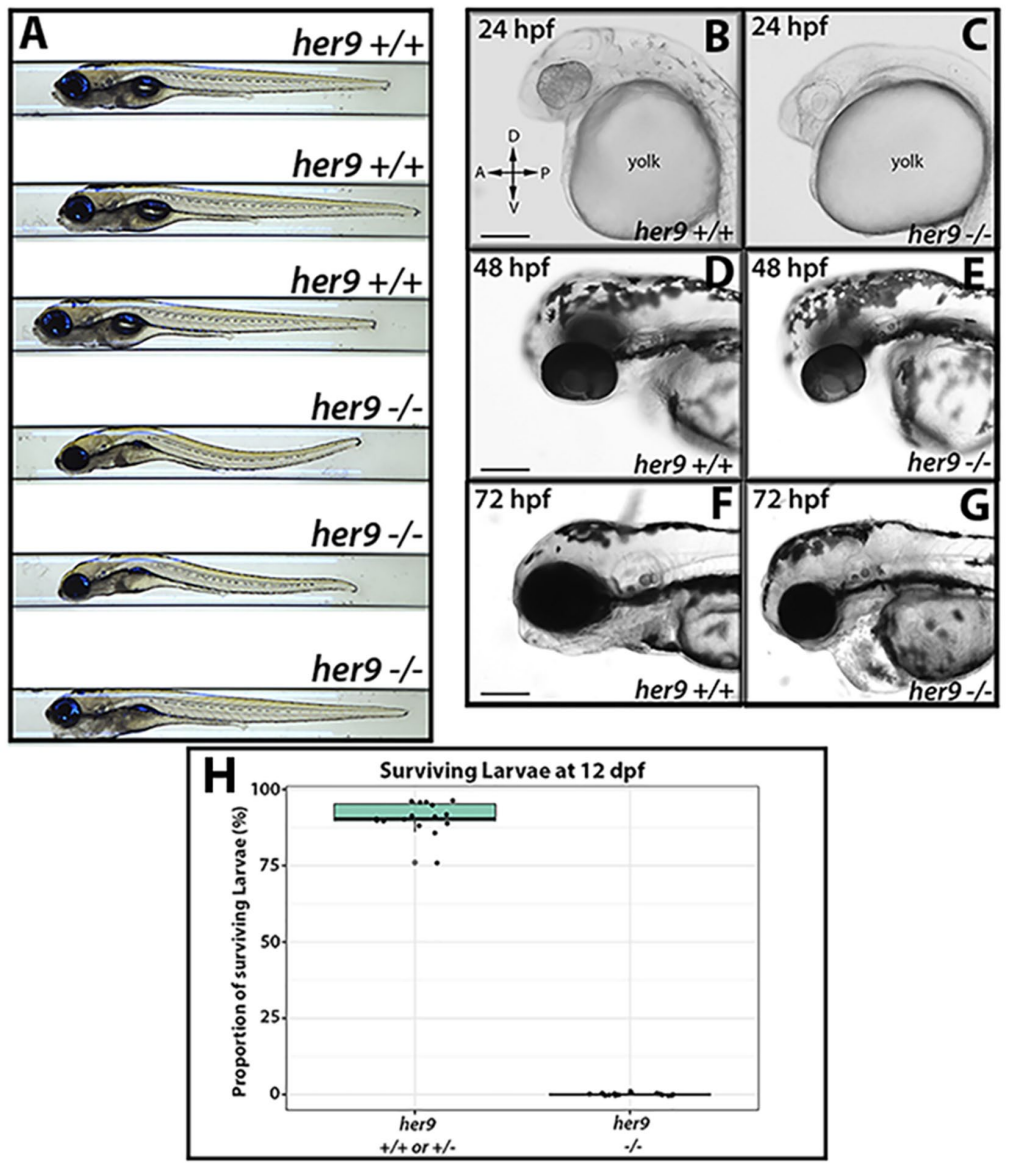
*Her9* mutant phenotype. (**A**) Gross morphology of WT and *her9* mutants at 5 dpf. (**B**–**G)** Gross morphology of WT and *her9* mutants at 24, 48, and 72 hpf. (**H**) Proportion of surviving larvae at 12 dpf; each point represents a separate cross. Scale bar = 50 µm and 100 µm.

We first characterized the progeny of *her9* heterozygous incrosses by light microscopy. At 24 hpf, *her9* mutants appeared somewhat developmentally delayed with lighter pigmentation and a smaller body size when compared to wild type (WT) and heterozygous siblings (Fig. 2B–C). The mutants also displayed delayed development of the midbrain and hindbrain ventricles (Fig. 2B), which appeared around 36 hpf (Fig. S3A–C). At 48 hpf, mutant embryos were smaller in body size, microphthalmic, and some displayed pericardial edema (Fig. 2D–E). At 72 hpf, homozygous mutants displayed a phenotypic range from mild to severe, where some mutant embryos looked fairly normal, and others displayed similar defects to those seen at earlier time points (Fig. 2F–G). In wild type (WT) and *her9* heterozygotes, the swim bladder was fully developed by 5 dpf (Fig. 2A). In contrast, *her9* homozygous mutant larvae did not develop a swim bladder (Fig. 2A) and displayed an abnormal swimming pattern, although quantification of swimming distance and velocity was not significantly different (Fig. S2). Other mutant phenotypes observed at 5 dpf included an enlarged liver, curved body, and craniofacial defects (Fig. 2A). At 12 days post fertilization (dpf) we noticed a significant decrease in larval survival with approximately 29% mortality. None of the *her9*^*−/−*^ larvae survived beyond 13 dpf, indicating that loss of Her9 results in larval lethality (Fig. 2H).

To rule out off-target contributions to the mutant phenotype, we performed a *her9* mRNA rescue experiment. Full-length *her9* mRNA was microinjected into one-cell stage embryos from *her9* heterozygous in-crosses, then the embryos were imaged and scored for the earliest visible mutant phenotype, which was lack of a clearly defined midbrain-hindbrain boundary and a missing midcerebral vein (MeCV) at 24 hpf (imaged by fluorescence microscopy of the *fli1*:GFP transgene; Fig. S3A–C). Whereas only 5.9% of uninjected *her9* homozygous mutant larvae had a detectable MeCV at 24 hpf, 80% of the *her9* mutant embryos that received *her9* mRNA had a MeCV (Fig. S3C–D). We also observed a significant increase in the vasculature around the eye and heart in mRNA-injected mutant embryos, as well as a larger eye size (Fig. S3B–C). Combined, these results indicate that the phenotypes described herein are specifically due to the mutation of *her9*, and also that Her9 may be required for proper vasculature development.

### *Her9* mutants lack a visual background adaptation response and display visual dysfunction

We observed that at 5 dpf, a significant number of the progeny of *her9*^+*/−*^ incrosses (~ 27%) appeared more darkly pigmented after prolonged light exposure when compared to their siblings. This suggested that *her9* mutants may lack a visually mediated background adaptation (VBA) response, a neuroendocrine camouflage response that allows zebrafish to manipulate their melanin granules in response to light exposure to match their background. Several previous studies have shown that the VBA response depends on retinal input^19–21^, thus an absent VBA response can indicate visual impairment.

**Figure 3:**
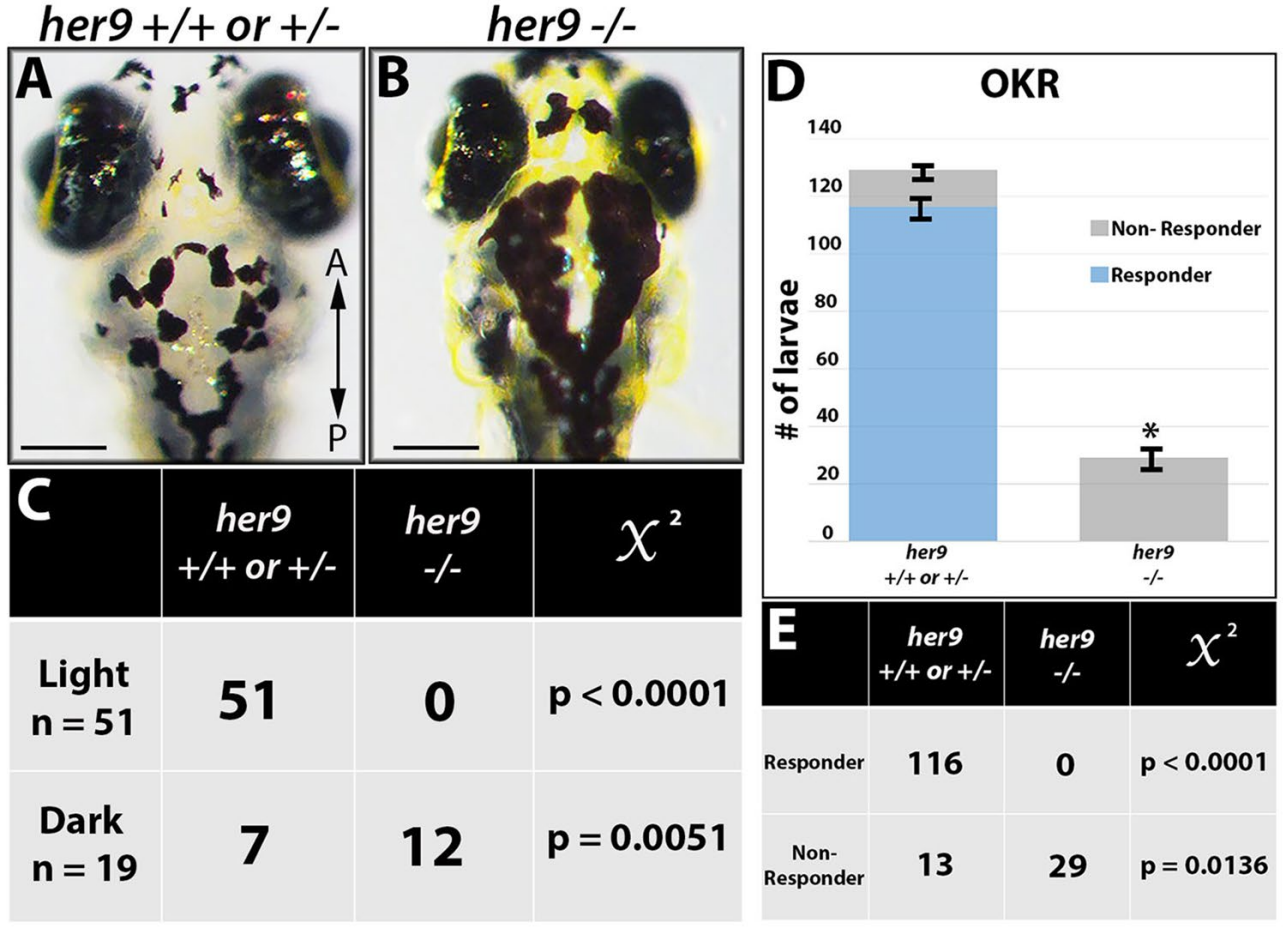
Her9 mutants lack a VBA response and display abnormal visual behavior (OKR). (**A**,**B**) Images displaying *her9* mutant with significantly darker pigmentation at 5 dpf. (**C**) Genotyping of the “dark” and “light” larvae revealed that out of 51 individuals classified as “light”, zero were *her9*^−/−^ (*p *value < 0.0001, χ^**2**^ analysis). Conversely, 12 out of 19 individuals classified as “dark” were *her9*^*-*−/−^ (*p* value = 0.0051). (**D**) Number of responders and non-responders in the optokinetic response (OKR) assay. (**E**) Genotyping after OKR revealed that zero of the *her9*^−*/*−^ embryos displayed saccades in the OKR (*p* < 0.0001). Scale bar = 50 µm.

To determine whether the lack of a VBA response was associated with the *her9* mutant genotype, we first dark-adapted the larvae for two hours, then placed them in ambient light for 30 min, imaged and scored them as dark or light, then extracted genomic DNA for genotyping. After light exposure, 51 out of 70 larvae were scored as light and 100% of those embryos genotyped as WT or heterozygous (*p* < 0.0001; Fig. 3A,C). In contrast, 19 of the larvae scored as dark, 63% of which genotyped as homozygous mutants (*p* = 0.0051; Fig. 3B–C). From these data, we conclude that *her9* mutants lack a normal VBA response, which could suggest visual impairment and possible retinal defects.

Given their lack of VBA response, we wondered whether the *her9* mutants had any functional visual deficit. To investigate this, we used the optokinetic response (OKR) assay, which is a behavioral test that measures the larvae’s ability to perform a combination of smooth pursuit and rapid saccade eye movements in response to a moving pattern of alternating black and white vertical stripes^22^. Individual larvae from a *her9* heterozygous incross were screened at 5 dpf over three 30-s trials (n = 159), and then genotyped after screening. Whereas 116 out of 129 WT and heterozygous larvae displayed a positive OKR response, none of the *her9* mutants responded with saccadic movements in the OKR assay (0 out of 29 tested; *p* < 0.0001; Fig. 3D–E). We conclude from these data that the loss of Her9 causes impaired visual responses at 5 dpf.

### *Her9* mutants display a decrease in rod photoreceptors and rod outer segment defects

**Figure 4:**
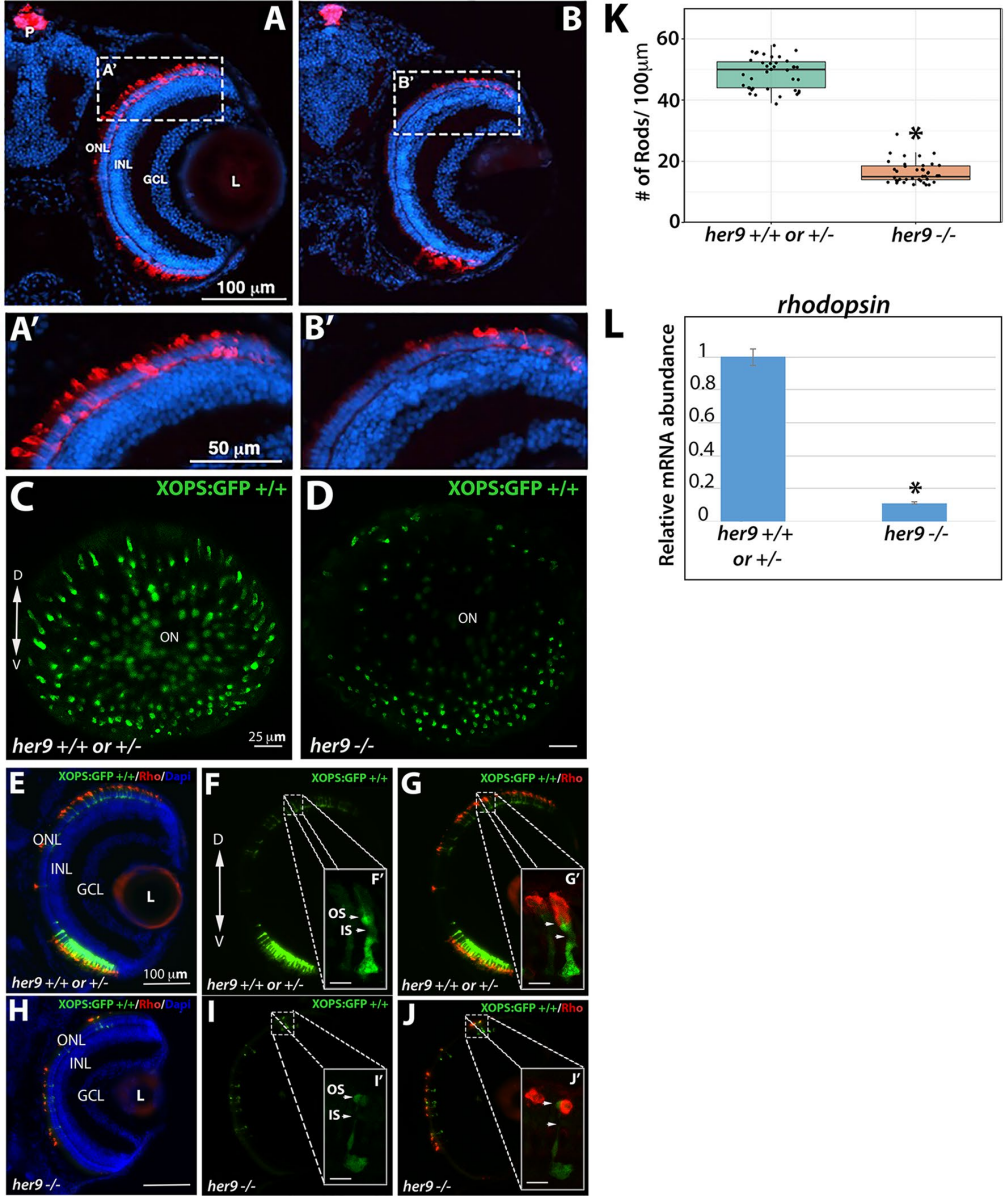
*Her9* mutants have fewer rods with abnormal outer segments. Immunohistochemistry with a rod antibody (4C12) in *her9*^+/+^ or ^+/−^ (**A**,**A′**) and *her9*^−/−^ (**B**,**B′**) retinal sections. (**C**–**D**) Confocal images of whole eyes from *her9* heterozygous incross progeny on the XOPS:GFP background. (**E**–**G**) Immunohistochemistry with an antibody that labels rhodopsin (1D1) on retinal cryosections of XOPS:GFP WT and heterozygous (**E**–**G**) or *her9* mutant (**H**–**J**) larvae. (**K**) Rod cell counts in *her9*^*−/−*^ larvae and their WT and heterozygous siblings (# of rods/100 µm). WT/Het = 10 embryos; Mut = 10 embryos; t-test (*p* < .0001). (**L**) qPCR analysis of *rhodopsin* expression at 8 dpf (fold change relative to *Ef1*α). ONL, outer nuclear layer; OPL, outer plexiform layer; INL, inner nuclear layer; GCL, ganglion cell layer; L, lens; ON, optic nerve; P, pineal gland. Scale bar = 50 µm and 100 µm.

Next, we used immunohistochemistry (IHC) on retinal tissue sections to examine the number and morphology of various retinal cell types in WT and *her9* mutant larvae, starting with the rod photoreceptors. Using a rod photoreceptor specific antibody (4C12), we observed numerous rod photoreceptors in the dorsal and ventral portions of the WT retina at 12 dpf (Fig. 4A). At higher magnification the rod outer segments were easily detected (Fig. 4A′). In contrast, *her9* mutant retinas displayed a significant decrease in rod photoreceptors which was especially apparent in the dorsal retina (Fig. 4B). At higher magnification we also observed a marked decrease in antibody labeling of the outer segments of the remaining *her9* mutant rods compared to the WT retina.

To more thoroughly examine the effects of the *her9* mutation on rod photoreceptor number and morphology, *her9* heterozygotes were crossed onto the XOPs:GFP transgenic background, which fluorescently labels rod photoreceptors^23^. The *her9* heterozygotes on the XOPs:GFP background were in-crossed, and whole eyes were removed from larvae at 5 dpf and imaged by confocal microscopy, while the rest of the body was used for genotyping. Using this approach, we observed a significant decrease in GFP^+^ rod photoreceptors across the entire *her9* mutant retina when compared to the WT (Fig. 4C–D), confirming our previous results.

The remaining heads were pooled together based on genotype and cryo-sectioned, followed by counting of GFP+ cells. We observed a 69% reduction in the number of GFP+ rods in *her9* mutant larvae compared to WT (avg. 18 per 100 µm in *her9* mutants vs. 58 per 100 µm in WT), which was confirmed via qPCR for *rhodopsin* (Fig. 4K–L). Finally, we used the 1D1 antibody to label the rod outer segments in WT and *her9* mutant retinal cryosections. In the *her9* mutant embryos 1D1 labeling of outer segments was severely reduced compared to the WT embryos (Fig. 4E–G′, H–J′). Taken together, these data demonstrate that loss of Her9 causes a large decrease in rod photoreceptor number and abnormal rod photoreceptor outer segment morphology.

### Cone outer segments are truncated in *her9* mutants

**Figure 5:**
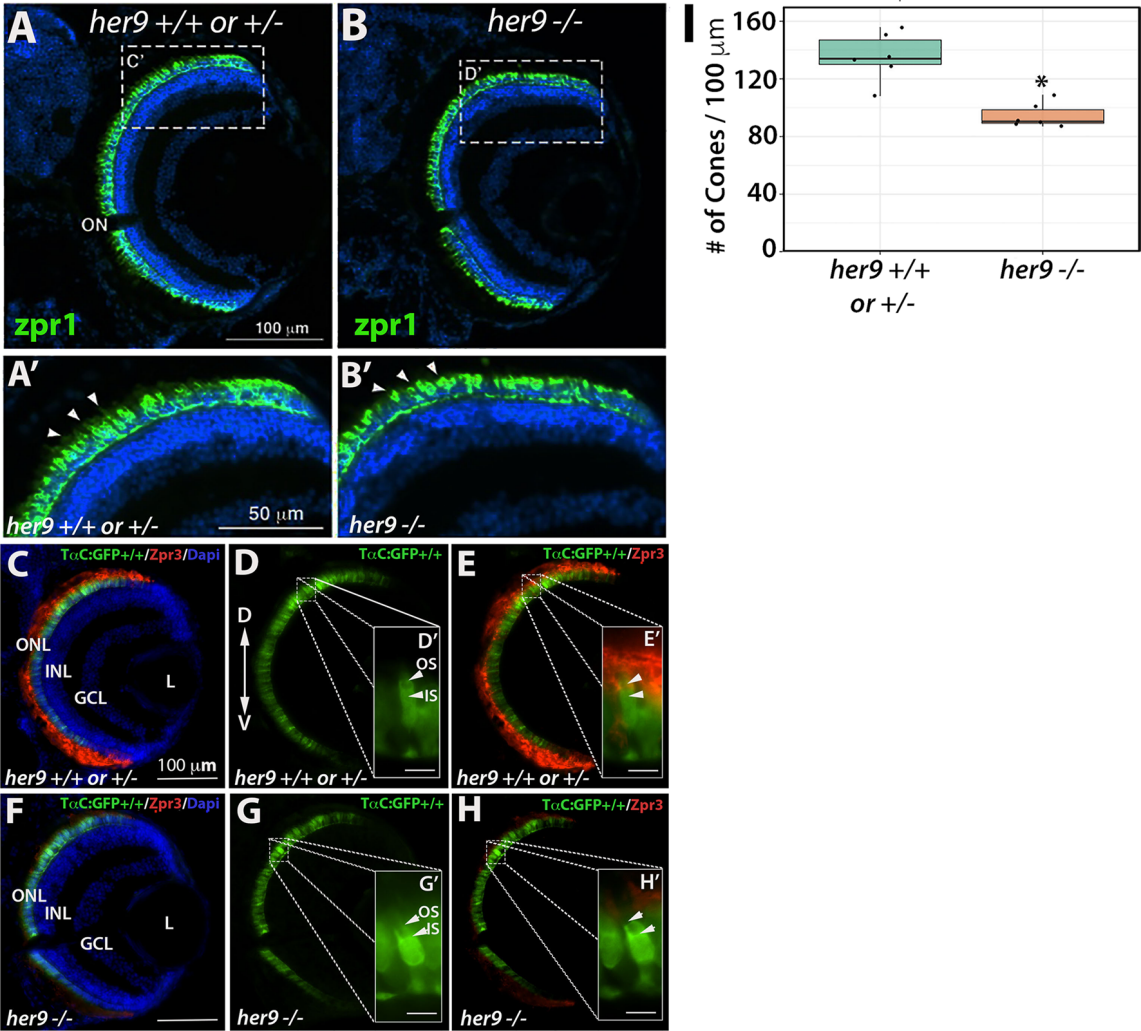
Cone outer segments are abnormal in *her9* mutants. Immunohistochemistry with a red-green cone antibody (Zpr1) in *her9*^+/+^ or ^+/*−*^ (**A**,**A′**) and *her9*^−/−^ (**B**,**B′**) retinal sections. Arrowheads indicate outer segments. (**C**–**H**) Immunohistochemistry with an antibody that labels rod and double cone outer segments (Zpr3) on retinal cryosections of TαC:GFP; *her9*^+*/*+^ or ^+*/−*^ (**C**–**E**) or TαC:GFP; *her9*^*−/−*^ mutant (**F**–**H**) larvae. **(I)** Cone cell counts in *her9*^*−/−*^ mutant larvae and their WT and heterozygous siblings (# of Cones/100 µM). WT/Het = 10 embryos; Mut = 10 embryos; t-test (*p* < .0001). ONL, outer nuclear layer; OPL, outer plexiform layer; INL, inner nuclear layer; GCL, ganglion cell layer; L, lens; ON, optic nerve; OS, outer segment; IS, inner segment. Scale bar = 100 µm.

We used the Zpr1 antibody to detect the red–green double cones in WT and *her9* mutant retinas. At 12 dpf, we initially did not observe an obvious decrease in cone number in the *her9* mutant retina (Fig. 5A–B). However, at higher magnification, we detected gaps in the spacing of the double cones, as well as shorter cone outer segments, in *her9* mutant retinas (Fig. 5A′–B′). To examine this further, we crossed the *her9* mutation onto the TαC:GFP transgenic background, which fluorescently labels all cone photoreceptor subtypes^24^; (Fig. 5C–H). There was a significant decrease in the number of cone photoreceptors in the *her9* mutants compared to WT (Fig. 5I). We used the Zpr3 antibody to label the outer segments of the double cones and rods, and found a noticeable reduction in antibody labeling in *her9* mutant retinas (Fig. 5C–H). These results demonstrate that the loss of Her9 causes a decrease in the number of double cones, and has profound effects on red and green cone outer segment morphology.

### *Her9* mutants display cone subtype-specific phenotypes

**Figure 6:**
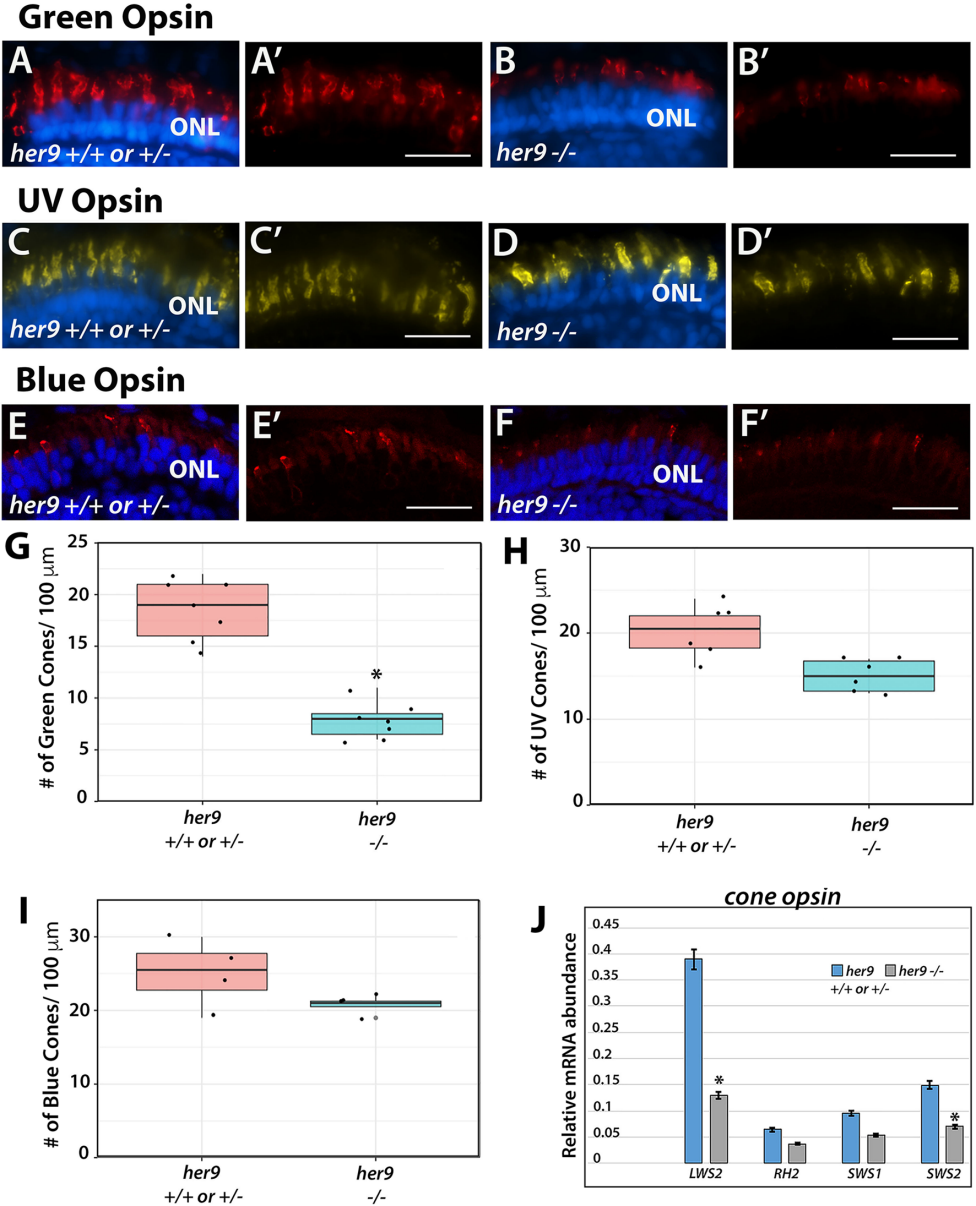
*Her9* mutants display cone subtype-specific phenotypes. Immunohistochemistry using a green (**A**–**B′**), UV (**C**–**D′**), or blue (**E**–**F′**) cone opsin antibody on WT and *her9* mutant retinal cryosections. (**G–I**) Cell counts of opsin expressing cells in *her9* mutant retinas compared to their siblings (# of opsin+ cells/100 µm). Green Opsin: WT/Het = 10 embryos; Mut = 10 embryos; t-test (*p* < .0004). UV Opsin: WT/Het = 10 embryos; Mut = 10 embryos; t-test (*p* < .0046). Blue Opsin: WT/Het = 10 embryos; Mut = 10 embryos; t-test (*p* = .1892). (**J**) qPCR analysis of the different cone opsins at 8 dpf (fold change relative to *Ef1α*). ONL, outer nuclear layer. Scale bar = 50 µm.

We next investigated whether all cone subtypes were equally affected by the loss of Her9, using cone opsin-specific antibodies to perform IHCs on retinal cryosections as described above. We again observed a significant reduction (42%) in the number of green cone photoreceptors and truncated green cone outer segments in the *her9* mutant retinas compared to their WT siblings (Fig. 6A–B,G). Interestingly, IHC with the UV- and blue-cone opsin antibodies revealed a much smaller decrease in number for those cone subtypes in *her9* mutant retinas compared to the decrease in green cones (6.6% and 5%, respectively; Fig. 6C–F′, H and I) and we did not detect truncation in the blue and UV cone outer segments (Fig. 6D′–F′). qPCR for long-, medium- and short-wavelength opsins confirmed a small decrease in UV- and blue- cone opsin expression and a large decrease in red cone opsin expression (Fig. 6J). Green cone opsin expression was also modestly reduced by qPCR. The results of these experiments suggest that loss of Her9 causes variable reductions in all cone photoreceptors and defects in outer segments that are specific to red/green cones and rods.

### Müller glia abnormalities in *her9* mutants

**Figure 7:**
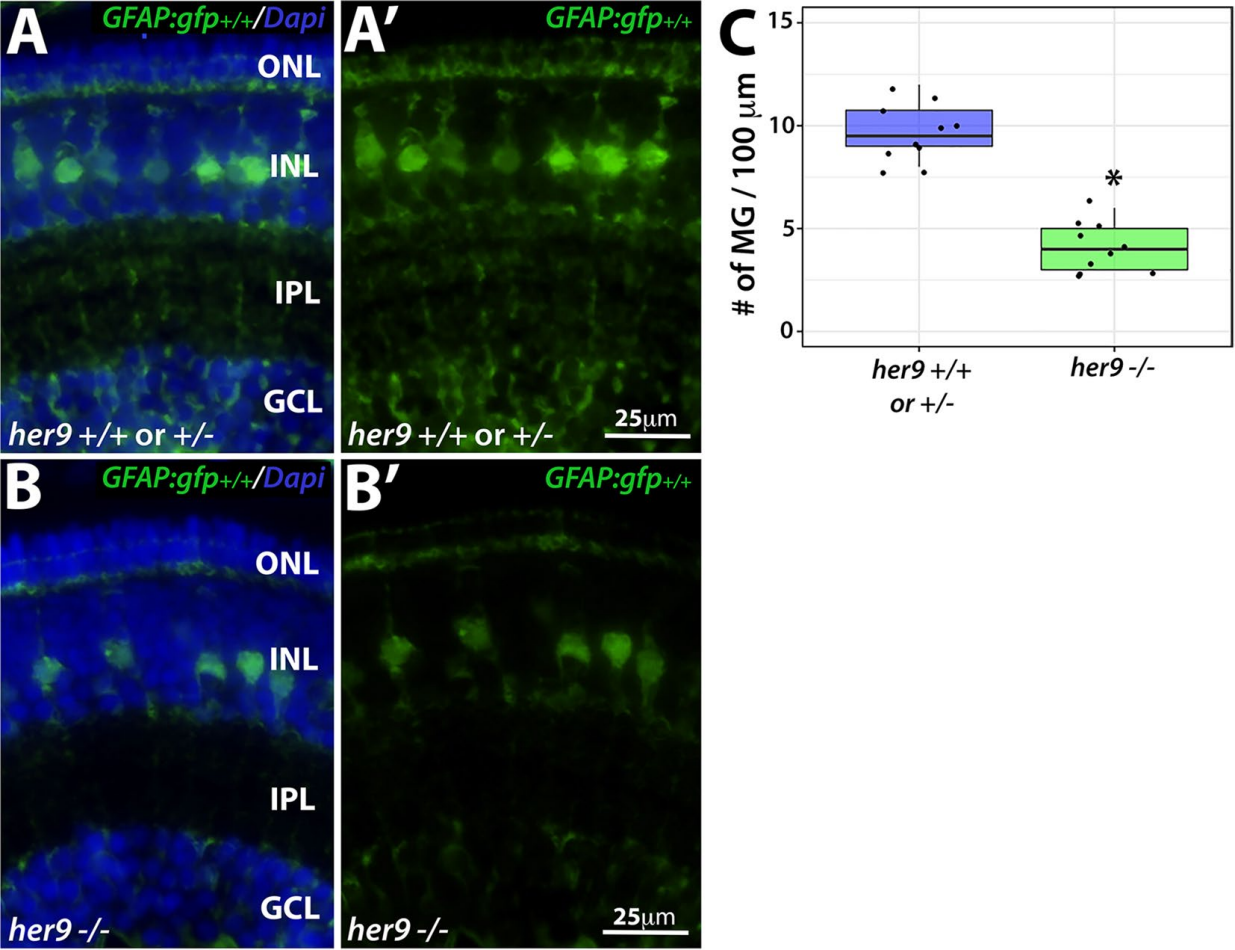
Loss of *her9* causes a decrease in Müller glia number and distorts their organization. Cryosections of WT and heterozygous (**A**–**A′**) or *her9* mutant (**B**–**B′**) retinas at 5 dpf on the *gfap*:GFP transgenic background. (**C**) Cell counts of GFP+ Müller glial cells in *her9* mutant retinas compared to their WT siblings (# of MG/100 µm). WT/Het = 10 embryos; Mut = 10 embryos; t-test (*p* < .0001).

To determine whether loss of Her9 affects other late-born cell types, we crossed the *her9* mutation onto the *gfap*:GFP transgenic background, which fluorescently labels retinal Müller glia^25^. At 5 dpf, *her9* homozygous mutants showed a significant reduction of GFP+ Müller glia compared to their WT and heterozygous siblings (*p* < 0.0001; Fig. 7A–C). We also observed a decrease in GFP+ glial cells across the entire *her9* mutant embryo (brain and enteric nervous system) at this stage (Fig. S4A–D′). In addition, the GFP+ Müller glia that were present in *her9* mutant retinas displayed a disorganized pattern, with their cell bodies located at various depths of the INL layer, rather than forming a uniform row as in the WT retinas (Fig. 7A′,B′). We also observed a lack of processes from the Müller glia spanning the retina in the *her9* mutants compared to their WT siblings. These data indicate that the loss of Her9 not only causes a reduction in the number of Müller glia, but also distorts the morphology, organization and patterning of the Müller glial cells that do develop.

### Loss of Her9 has minimal effects on other retinal cell types

**Figure 8:**
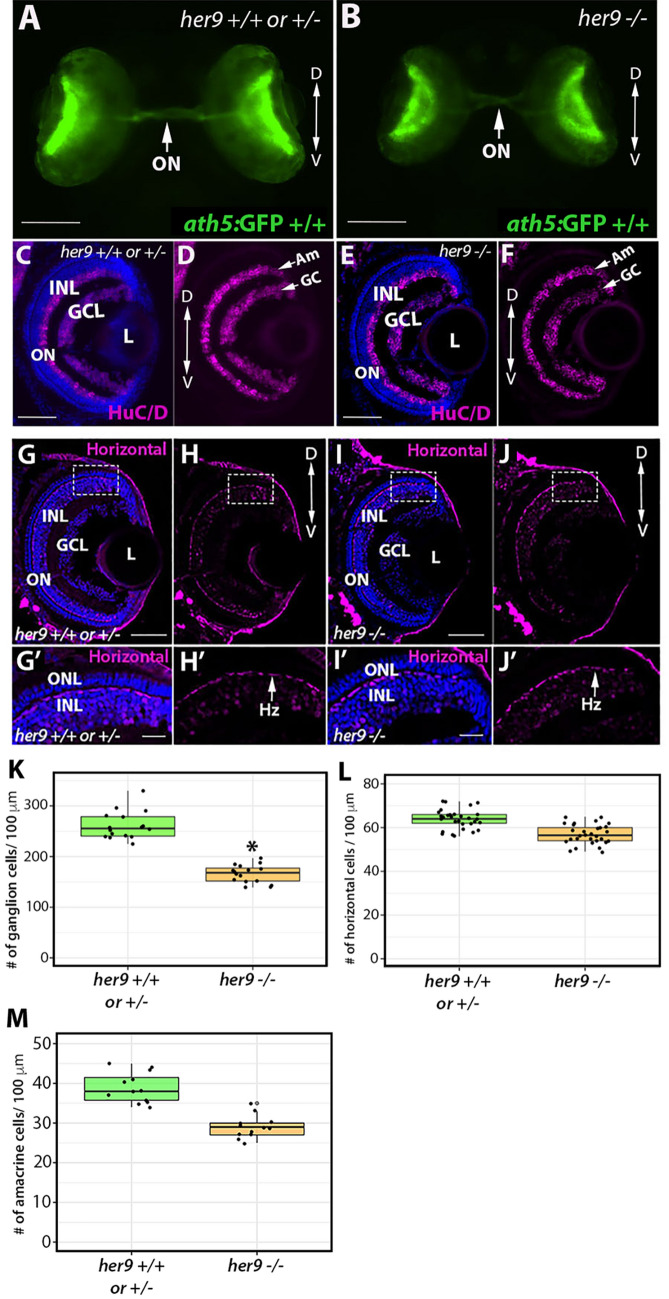
Loss of *her9* has minimal effects other retinal cell types. Whole eye images of WT and heterozygous embryos (**A**) or *her9* mutant embryos (**B**) on the Ath5:GFP transgenic background at 5 dpf. Immunohistochemistry for ganglion and amacrine cells (HuC/D antibody) in cryosections from WT and heterozygous retinas (**C**–**D**) or *her9* mutant retinas (**E**–**F**). Ganglion cell**:** WT/Het = 10 embryos; Mut = 10 embryos; t-test (*p* < .0001). Horizontal cells: WT/Het = 10 embryos; Mut = 10 embryos; t-test (*p* = .455). Amacrine cells: WT/Het = 10 embryos; Mut = 10 embryos; t-test (*p* = 0.5). (**G**–**H′**) Immunohistochemistry with the Prox1 antibody labels horizontal cells in cryosections of WT and heterozygous (**G**–**H′**) or *her9* mutant (**I**–**J′**) retinas. Cell counts reveal a decrease in the number of ganglion cells in the mutant retinas (**K**) but no significant difference in the number of horizontal or amacrine cells (**L-M**). Am, Amacrine cells; GC, Ganglion cells; Hz, Horizontal cells; ONL, outer nuclear layer; INL, inner nuclear layer; ON, optic nerve; L, Lens. Scale bar = 50 µm and 100 µm.

Does the loss of Her9 disrupt development of all retinal cell types? To address this question, we used transgenic lines or IHC with cell-type specific antibodies for ganglion cells, amacrine cells, and horizontal cells. First, we crossed *her9* heterozygous zebrafish onto the ath5:GFP transgenic background^26^ which fluorescently labels ganglion cells and the optic nerve (Fig. 8A–B). We observed a modest decrease in GFP expression in the *her9* mutant retinas compared to WT (Fig. 8B; arrow). We followed this up with IHC using the HuC/D antibody, which labels both ganglion cells and amacrine cells. We observed a significant decrease in the number of ganglion cells in *her9* mutant retinas compared to the WT and heterozygous siblings (Fig. 8C–F, K; *p* < 0.0001). However, the number of amacrine cells was not significantly different in the WT versus *her9* mutant retinas at 5 dpf (Fig. 8M).

The Prox1 antibody was then used to assess horizontal cells in the retinas of *her9* ± in-cross progeny (Fig. 8G–J′). There were no morphological differences in the horizontal cells of *her9* mutants compared to WT (Fig. 8H′, J′), and the number of horizontal cells was not significantly reduced (Fig. 8L). These results demonstrate that loss of Her9 causes a mild decrease in ganglion cell number, but does not alter the numbers of amacrine or horizontal cells, and does not disrupt the morphology of these retinal cell types. This indicates that the morphological defects due to loss of Her9 are specific to photoreceptors and the Müller glia.

### Loss of Her9 causes a progressive collapse of the CMZ

**Figure 9:**
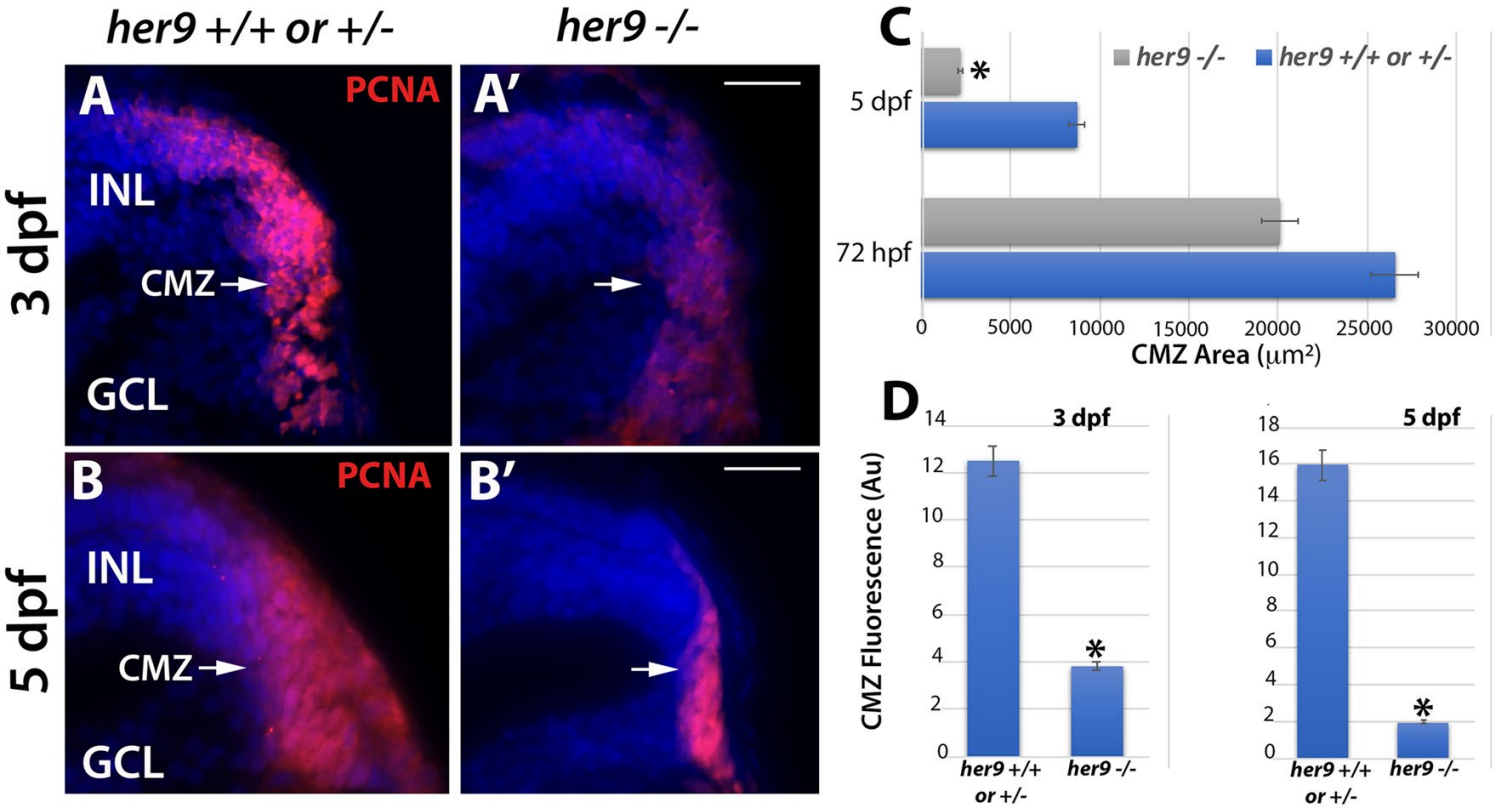
CMZ defects in *her9* mutants. Immunostaining for PCNA in WT or heterozygous (**A**,**B**) and *her9* mutant (**A′**,**B′**) retinal sections at 72 hpf and 5 dpf; a decrease in the size and fluorescence intensity of the CMZ is apparent in *her9* mutants. Area of CMZ (defined as the region of PCNA+ cells directly adjacent to the end of the inner plexiform layer) calculated at 72 hpf and 5 dpf (**C**). *Her9* mutants have a significantly reduced PCNA-positive signal in the peripheral retina. CMZ fluorescence intensity at 72 hpf and 5 dpf (**D**). GCL, ganglion cell layer; INL, inner nuclear layer; CMZ, ciliary marginal zone. Scale bar = 50 µm.

Previous studies of the post-embryonic Medaka and Xenopus retina showed that *her9* regulates the proliferation of the retinal stem cells in the CMZ^17^. Therefore, we wanted to investigate whether germline mutation of zebrafish *her9* would affect the establishment of the CMZ during retinal development. We collected zebrafish larvae at 72 hpf, then prepared retinal sections for PCNA immunolabeling, which detects cells in S-phase (Fig. 9A–A′). We observed a significant decrease in the area of PCNA+ cells in the CMZ (defined as the region directly adjacent to the end of the inner plexiform layer) of mutant retinas in comparison to their WT and heterozygous siblings. To determine whether the *her9* mutant CMZ could recover to a normal size later in development, we immunolabeled 5 dpf retinal sections with the PCNA antibody. We again observed a significantly smaller PCNA+ CMZ in 5 dpf *her9* mutant retinas compared to their WT and heterozygous siblings (Fig. 9B–B′). Quantification of both the PCNA-labeled CMZ area and fluorescence intensity confirmed a significant decrease in the *her9* mutants compared to WT and heterozygous siblings (Fig. 9C–D). Interestingly, the lack of proliferating cells in the CMZ became more severe from 72 hpf to 5 dpf in *her9* mutants, indicating that there is a decrease in the number of proliferating cells in the mutant CMZ over time. Taken together, our results confirm that Her9 regulates the proliferation or maintenance of stem cells in the CMZ.

### *Her9* mutants display abnormal expression of photoreceptor lineage genes

**Figure 10:**
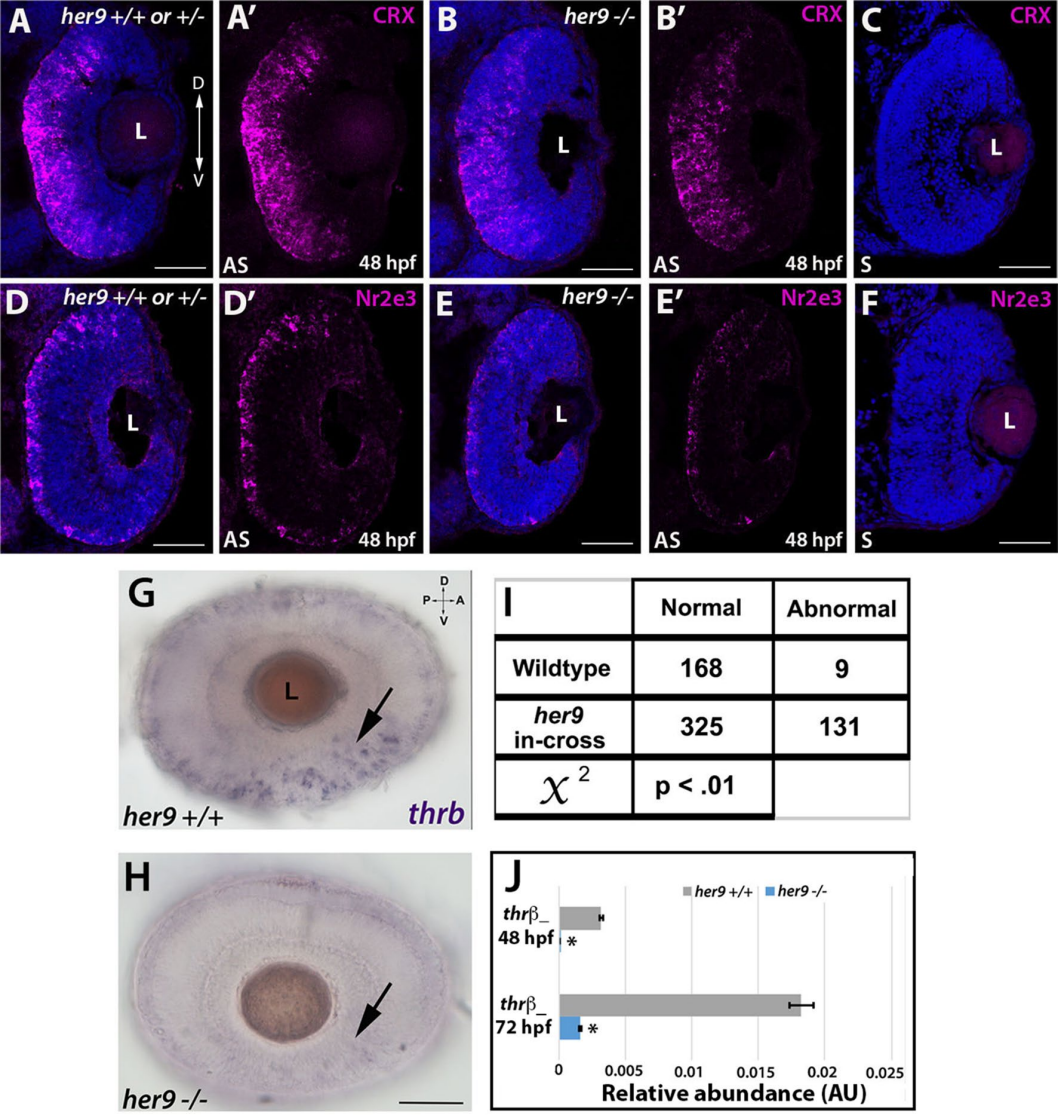
*Her9* mutants display abnormal expression of photoreceptor lineage genes. (**A**–**B**′) Fluorescent in situ hybridization (FISH) for *crx* at 48 hpf in WT and mutant neural retina. (**C**) *Crx* sense probe. (**D**–**E′**) Decreased expression of *Nr2e3* in the mutant retina compared to the wildtype. (**F**) *Nr2e3* sense probe. (**G**,**H**) Whole mount in situ hybridization (WISH) for *thrβ* expression at 48 hpf in WT and *her9* mutant retina. *thrβ* expression in *her9* mutants displayed a decreased and patchy or abnormal pattern. (**I**) χ^2^ analysis comparing WT incross and *her9* heterozygous incross expression pattern. (**J**) qPCR for *thr*β at 48 and 72 hpf. L, lens. Scale bar = 50 µm.

To begin to address the mechanism underlying the photoreceptor phenotypes of *her9* mutant retinas, we first examined whether loss of *her9* affects the specification of retinal progenitor cells towards rod and cone lineages. Crx is a transcription factor that plays a critical role in the specification of all photoreceptor subtypes^27^. We used FISH on retinal cryosections to examine the expression of *crx* at 48 and 72 hpf. Starting at 48 hpf, the expression of *crx* spreads across the WT retina in a ventral to dorsal fan-like manner (Fig. 10A–A′). In *her9* mutant retinas we observed a similar expression pattern of *crx*, albeit with reduced signal intensity (Fig. 10B–B′); by 72 hpf, *crx* expression in *her9* mutant retinas was not significantly different from WT (Supplemental Fig. S5A–B′). Control sense probes showed no signal (Fig. 10C,F). Therefore, we conclude that loss of Her9 does not perturb specification of photoreceptor progenitors.

Next, we examined expression of photoreceptor subtype-specific transcription factors. Nr2e3 is responsible for the activation of rod photoreceptor genes and repression of cone genes in photoreceptor progenitors^28^. At 48 hpf in the WT retina, we observed robust expression of *Nr2e3* throughout the ONL of the retina (Fig. 10D–D′). In contrast, in *her9* mutants, there was significantly less *Nr2e3* expression within the ONL in comparison to the WT (Fig. 10E–E′). At 72 hpf the expression of *Nr2e3* in the *her9* mutant retina had increased relative to 48 hpf, but the expression pattern remained disorganized relative to WT (Fig. S5C–D′). Taken together, this result indicates that the rod photoreceptors in *her9* mutants are specified but their differentiation is abnormal.

Next, we investigated whether the red cone photoreceptor lineage was disrupted in *her9* mutants. To do this, we used whole mount in situ hybridization (WISH) to investigate the expression of *trβ2* in the developing retina at 48 and 72 hpf in progeny from WT and *her9* heterozygous incrosses. The eyes were then removed from the embryos for imaging by light microscopy. In WT larvae, most of the individuals displayed robust expression of *trβ2* in the ventral portion of the eye and along the ONL circumference where the cone photoreceptors are located (the “normal” pattern), although a few WT larval eyes (5.3%) showed fainter expression in the ventral portion of the eye and in the ONL (n = 9 out of 177; Fig. 10G–H). In contrast, 28.7% of the larval eyes from *her9* heterozygous incrosses (n = 131 out of 456) displayed a decrease in the expression of *trβ2* and patches of missing expression around the circumference of the retina, representing an “abnormal pattern” for *trβ2* expression (χ^2^ analysis, *p* < 0.01; Fig. 10I). qPCR analysis at 48 hpf and 72 hpf confirmed the reduced expression of *trβ2* in *her9* mutants compared to their wildtype siblings (Fig. 10J). Therefore, we conclude that loss of Her9 disrupts the specification of the red cone lineage.

### *Her9* mutant photoreceptors undergo apoptosis

**Figure 11:**
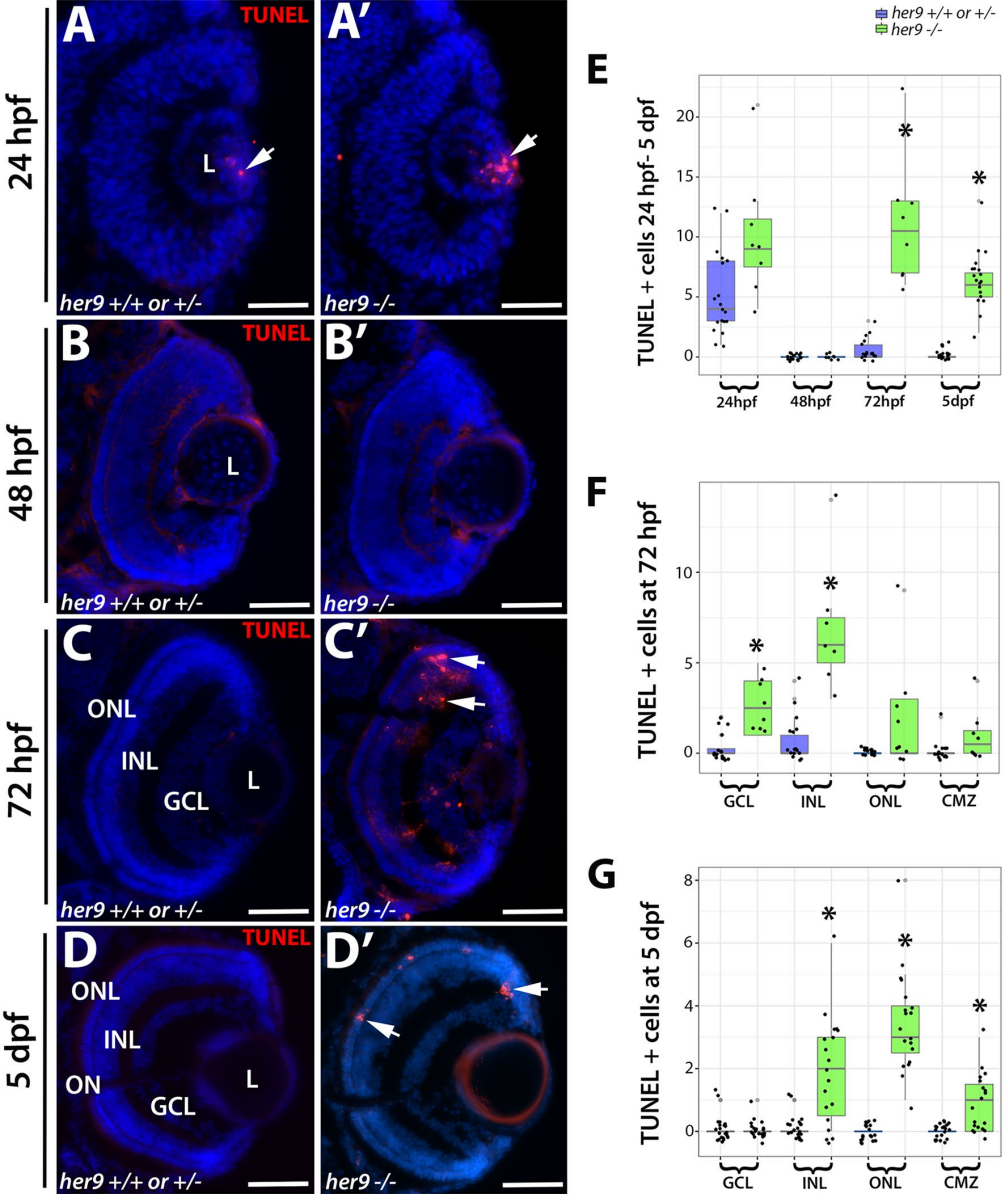
Her9 mutant retinas display increased apoptosis beginning at 72 hpf. (**A**–**D′**) TUNEL staining on cryosections of WT and *her9* mutant retinas**.** *Her9* mutant embryos show no significant increase in cell death in the retina at 24 and 48 hpf compared to WT siblings (**A**–**B′**). At 72hpf, *her9* mutant larvae display increased apoptosis in the GCL, INL, ONL and CMZ (**C**–**C′**) that remains elevated in the ONL at 5 dpf (**D**–**D′**). Cell counts of TUNEL+ cells (**E**–**G**). Note that the data points below the x-axis do not indicate negative numbers, but rather are an attempt by the ggplot2 package to display all the data points (ggplot2 in R: version 3.6.2; https://www.R-project.org/). ONL, outer nuclear layer; INL, inner nuclear layer; GCL, ganglion cell layer; L, lens. Scale bar = 50 µm.

In addition to abnormal specification, the reduced numbers of rods and double cones in *her9* mutants could reflect a decrease in survival of differentiated photoreceptors. To determine whether this was the case, we performed TUNEL labeling on retinal cryosections from WT and *her9* mutant retinas from 24 hpf to 5 dpf to identify apoptotic cells. At 24 hpf, we observed a slight increase in TUNEL+ cells in the lens of *her9* mutants compared to WT, but no cell death in the retina (Fig. 11A–A′, E). At 48 hpf, there was no difference in TUNEL+ cells between WT and *her9* mutant retinas (Fig. 11B–B′, E). However, at 72 hpf we observed a significant increase in TUNEL+ cells in the GCL, INL, ONL, and CMZ of *her9* mutant retinas compared to the WT (Fig. 11C–C′, E, F). Interestingly, we observed a noticeable cluster of TUNEL+ cells in the dorsal peripheral retina adjacent to the CMZ (Fig. 11C′). Additionally, some of the TUNEL+ cells in the INL had the morphology of Müller glia, which could indicate the Müller glia were dying, but could also be due to Müller glia phagocytosis of other dying cells (Fig. 11C′). By 5 dpf, apoptosis in *her9* mutants remained elevated in the ONL, CMZ, and INL relative to WT retinas (Fig. 11D–D′, G). These data indicate that Her9 is required for the survival of post-embryonic retinal progenitor cells, photoreceptors, and possibly cells in the INL.

### RA regulates *her9* expression in the retina but Her9 is not required for RA’s effects on opsin expression

**Figure 12:**
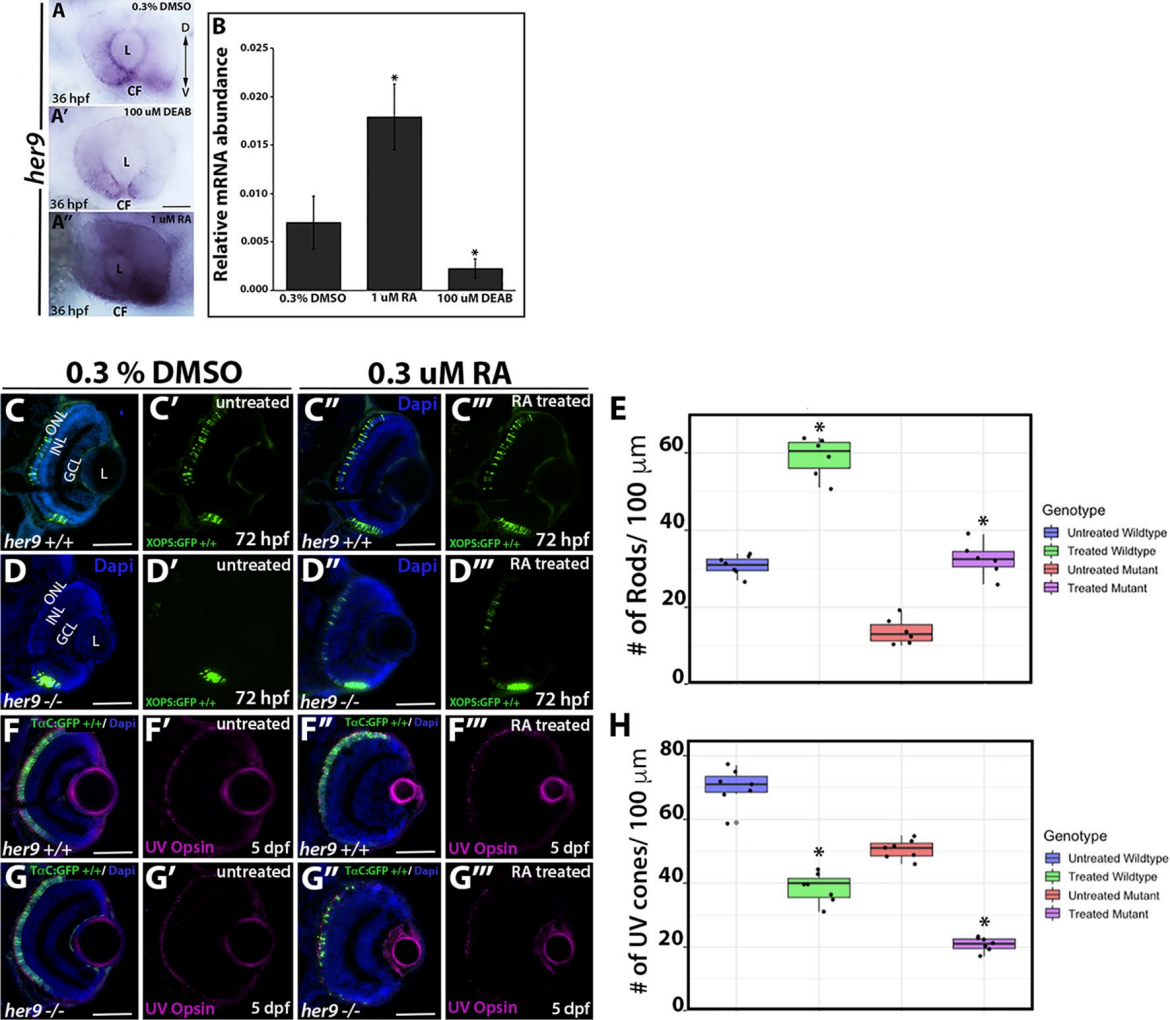
RA regulates *her9* expression but Her9 is not required for the effects of RA on opsin expression. WISH for *her9* expression at 36 hpf in control treated (**A**), DEAB treated (**A′**) and RA treated (**A″**) retinas. (**B**) qPCR for *her9* expression in heads of 36 hpf zebrafish embryos following drug treatments. N = 20 heads per biological replicate, 3 biological replicates per drug treatment. *p < 0.05. GFP expression in control treated versus RA-treated XOPs:GFP; *her9*^*+/+*^ (**C**–**C′″**) or XOPs:GFP; *her9*^−/−^ (**D**–**D′″**) retinas. (**E**) Cell counts comparing rhodopsin expressing cells in the untreated and treated retinas. (**F**–**F′″**) IHC for UV opsin in control versus RA-treated TαC:GFP; *her9*^+/+^ (**F**–**F′″**) or TαC:GFP; *her9*^*−/−*^ (**G**–**G′″**) retinas. (**H**) Cell counts comparing UV opsin expressing cells in the untreated and treated retinas (# of opsin+/100 µm). CF, choroid fissure; ONL, outer nuclear layer; INL, inner nuclear layer; GCL, ganglion cell layer; L, lens; Scale bar = 50 µm.

What regulates Her9 activity in the retina? Several previous studies have demonstrated that unlike many Hes/Hey/Her family members, Her9 does not respond to Notch signaling^10,15,17^. However, it has been shown that Her9 is downstream of RA signaling in the developing inner ear^9^. Given that RA signaling is critical for eye development^29,30^ and has demonstrated effects on photoreceptor differentiation and opsin expression ^31–33^, we hypothesized that Her9 functions downstream of RA to regulate opsin expression and photoreceptor survival. We used in situ hybridization and qPCR on 36 hpf embryos to determine whether *her9* expression in the retina is altered by manipulation of the RA signaling pathway. Zebrafish embryos were treated in the dark from 24 to 36 hpf with 1 µM RA, 100 µM of the RA signaling inhibitor diethylaminobenzaldehyde (DEAB), or 0.3% DMSO alone as a carrier control. Embryos were then processed for WISH or qPCR to detect *her9* expression. In control embryos, *her9* expression was observed in the peripheral CMZ and around the lens and choroid fissure as described above (Fig. 12A). We observed a noticeable decrease in *her9* expression in the retina when embryos were treated with DEAB (Fig. 12A′). In contrast, there was a significant increase in the expression of *her9* all across the retina when embryos were exposed to RA (Fig. 12A″). qPCR analysis for *her9* expression confirmed the decrease and increase in *her9* expression following DEAB and RA treatments, respectively (Fig. 12B). From these data, we conclude that *her9* expression in the retina is regulated by RA signaling.

Previous work demonstrated that exposure to exogenous RA between 2 and 5 dpf resulted in increased rhodopsin expression and decreased red, UV, and blue cone opsin expression in zebrafish^34^. To test the hypothesis that Her9 is required to mediate the effects of RA signaling on photoreceptor opsin expression, we treated WT and *her9* mutant zebrafish with RA from 24 to 48 hpf (for rods) or 24 hpf to 5 dpf (for cones), and then examined XOPS:GFP or TαC:GFP transgene and cone opsin expression by IHC. In WT zebrafish, RA treatment resulted in a significant increase in GFP+ rhodopsin expressing cells dorsally and ventrally compared to the untreated retinas, and a significant decrease in UV and green cone opsin expression, as expected (Fig. 12 and Fig. S6). Surprisingly, an increase in GFP+ rhodopsin expressing cells and a decrease in UV and green cone opsin expressing cells were also observed in RA-treated *her9* mutants when compared to control treated mutants (Fig. 12 and Fig. S6). Taken together, these data suggest that although *her9* expression in the retina is downstream of RA signaling, Her9 is not strictly required for the effects of RA on rod and cone opsin expression.

## Discussion

The zebrafish *her9* gene is the ortholog of the human *HES4* gene and is one of the least studied bHLH-O transcription factors. This is largely due to the fact that a Hes4 homolog is absent from the mouse and rat genomes^14^. Since *HES4* is expressed in humans and has been shown to play critical roles in the development of several vertebrate tissues, further investigations of Her9/HES4 function are needed. Indeed, the human HES4 locus lies within a critical region on the p arm of chromosome 1 that is frequently deleted in 1p terminal deletion syndrome, the most common chromosomal terminal deletion syndrome in humans, affecting ~ 1/5,000 newborns. Approximately 80% patients with this syndrome display eye malformations and/or visual impairment ^35,36^. Other phenotypes that we have observed in *her9* mutants are also consistent with some of the common features of 1p terminal deletion syndrome, such as developmental delay, craniofacial abnormalities, and growth and feeding problems. Therefore, it is possible that HES4 is a novel candidate gene contributing to phenotypes associated with this disorder. To our knowledge, ours is the first study to describe a germline loss of function mutation of *her9/HES4*, which we believe will be a powerful resource for elucidating its role in several developmental contexts.

In this study, we focused on the role of Her9 during retinal development. We observed *her9* expression predominantly in the ventral most portion of the developing retina at 24 hpf, which localized to the ciliary marginal zone (CMZ) around 36 hpf until at least 5 dpf (Fig. 1). This expression pattern is consistent with what has previously been described for *her9* in Xenopus and Medaka^14,17^, suggesting a conserved mode of action in the developing retina at least across fish and amphibians.

To characterize the role of Her9 in zebrafish development, we used CRISPR/Cas9 to generate loss of function mutations in *her9*. The mutations caused frame-shifts, and early termination codons, leading to nonsense-mediated decay of *her9* mRNA, which is supported by our sequencing, qPCR and Western blot data. The *her9* mutants appear developmentally delayed at around 20 hpf but by 72 hpf many *her9* mutant larvae look similar to their WT siblings. The relatively mild embryonic phenotype could be due to the buffering effects of maternally deposited WT *her9* mRNA. Nevertheless, by 5 dpf the mutants have not developed a swim bladder, display craniofacial and gastrointestinal defects, darker pigmentation, and abnormal swimming patterns. The *her9* mutants do not survive past 12 dpf, which may be due to problems with feeding and digestion.

One of the most striking phenotypes in the *her9* mutant consists of a significant decrease in rod and double cone photoreceptors, which is associated with elevated levels of apoptosis in the ONL and abnormal outer segments in the surviving rods and red/green cones. Intriguingly, this defect does not seem to affect the short wavelength (blue and UV) photoreceptor subtypes. The rod-cone dystrophy observed in *her9* mutants suggests that Her9 is required for rod and cone maintenance and survival. The degeneration of the rod photoreceptors seems more severe than the cones, supporting a rod-cone progression in degeneration. Given that we did not observe expression of *her9* in mature photoreceptors, this suggests that Her9 acts non-cell autonomously to maintain rods and double cones. Follow-up transplantation experiments could help to resolve this question. It is unlikely that the RPE contributes to the photoreceptor phenotypes, since we did not observe *her9* expression in the RPE, nor were there any obvious RPE structural defects in *her9* mutants. One possibility is that one of the functions of Her9 in the peripheral retina is to influence gradients of retinoic acid or thyroid hormone signaling, which are known to be important for photoreceptor maintenance (see below). In any case, the reduced number of cones and abnormal outer segments could explain the lack of VBA and OKR responses in the *her9* mutants, although we cannot rule out the possibility that the absent OKR response is due to defects in other tissues outside the retina. Given that we observed a significant decrease in rod and cone opsin expression, it is also possible that other phototransduction proteins are downregulated as well. Future electrophysiological and ultrastructural studies could help to resolve the functional consequences of Her9 loss to the photoreceptor outer segments.

The process of photoreceptor development has been extensively studied and important transcription factors that regulate photoreceptor specification have been identified, such as Crx, Nr2e3, and *thr*β, among many others. In our investigations of whether Her9 is required for rod and/or cone lineage specification, we saw no significant changes in *crx* expression in *her9* mutant retinas, indicating that Her9 is not required at this stage of photoreceptor specification. However, we did observe a significant decrease in expression of *Nr2e3* and *thr*β in *her9* mutants, indicating that Her9 may be required for the specification of some photoreceptor subtypes once progenitors have chosen a specific photoreceptor lineage.

The reduction in *thrβ* expression in *her9* mutants may contribute to additional phenotypes outside of the retina. Lui and Chan^37^ showed that thyroid hormones are important for the embryonic to larval transition in zebrafish. Inhibition of Thrβ produced a missing swim bladder, defects in the gastrointestinal tract, and craniofacial defects. These phenotypes resemble those observed in the *her9* mutants where we also see a loss of swim bladder, gastrointestinal tract defects, and craniofacial abnormalities in addition to the photoreceptor defects. Furthermore, given that there is a direct link between *thrβ* expression and cone development and differentiation^38^, our data suggest that Her9 could be directly or indirectly required for *thrβ* expression. This theory could be tested by exposing mutant embryos to L- thyroxine (T4) and 3,5,3′-l-triiodothyronine (T3) to see whether we can rescue any components of the *her9* mutant phenotype.

In addition to decreases in photoreceptors, we also observed significant decreases in the number of Müller glia, ganglion cells, and in proliferating cells in the CMZ of *her9* mutants. Investigation into the role of Her9/Hes4 in both Medaka and *Xenopus* have demonstrated expression of *her9* in the CMZ of the developing and post-embryonic retina^14,17^. Consistent with those data, we also observed the expression of *her9* localized to the CMZ starting at 36 hpf. We observed a significant decrease in the proliferating cells of the CMZ of *her9* mutants that became more severe over time. This indicates that Her9 is required for the maintenance of proliferating retinal progenitor cells. Morpholino knockdown of Hes4 in Xenopus produced dose-dependent eye defects from small eyes to animals with no eyes at all^14^. In our zebrafish *her9* mutants, we observed a variable degree of microphthalmia but never complete loss of eyes. This difference in phenotypic severity could be due to species-specific requirements for Her9 during oculogenesis, or to differences in experimental approach. In addition to a decrease in retinal cells, previous studies in *Xenopus* have shown that the loss of Hes4 leads to a significant increase in apoptosis in the retina^39–41^. In our model, we also observe a significant increase in apoptosis in the retina and brain of our *her9* mutant which leads us to conclude that Her9 plays a role in cellular proliferation early on in retinal development and then cell survival later.

Radosevic et al.^9^ demonstrated that Her9 is downstream of the Retinoic acid (RA) signaling pathway in the neural patterning that underlies the development of the otic vesicle. With these data in mind, we wanted to determine whether Her9 is also downstream of RA signaling in the developing retina. Accordingly, we observed an increase in *her9* expression in the retina following exogenous treatment with RA, and blocking RA signaling with DEAB resulted in a decrease in *her9* expression. These data demonstrate that *her9* expression is regulated by RA signaling in the developing zebrafish retina. Taken together with the outer segment defects and decreased opsin expression in *her9* mutants, as well as previous studies demonstrating that opsin expression is regulated by RA signaling^29–31^, we hypothesized that loss of Her9 was directly disrupting opsin expression downstream of RA signaling. However, when we exposed *her9* mutants to exogenous RA, we observed similar modulations in opsin expression to those observed on their WT siblings. This suggests that Her9 is not acting directly on opsin expression and may instead be acting downstream of RA to indirectly regulate photoreceptor differentiation and opsin expression. Future studies will include determining whether Her9 is interacting with other components of the RA pathway such as retinoic acid receptors, retinoid X receptors, or retinoic acid synthesis or degradation enzymes, some of which are expressed in the peripheral retina similarly to *her9*.

In summary, we have characterized a novel role for Her9 in photoreceptor development, maintenance, outer segment morphology and opsin expression, as well as Müller glial development. Given that the *her9* mutant displays such significant defects in rod and cone photoreceptors our results implicate Her9/HES4 as an important component of the gene regulatory network that regulates photoreceptor differentiation and survival. Further experiments such as cell lineage tracing are needed to resolve how much each component (cell differentiation, maintenance, and survival) contributes to the retinal phenotypes observed in *her9* mutants. Future studies of Her9/HES4 could add to our understanding of its role in regulating retinal stem cell proliferation at the ciliary margin and determine how its loss leads to photoreceptor degeneration.

## Materials and methods

### Zebrafish lines and maintenance

All zebrafish lines were bred, housed, and maintained at 28.5 °C on a 14 h light: 10-h dark cycle, in accordance with established protocols for zebrafish husbandry^42^. The Tg(3.2TαC: EGFP) transgenic line (hereafter called TαC:EGFP) has been previously described^24^, and was generously provided by Susan Brockerhoff (University of Washington, Seattle, WA). The Tg(gfap: GFP)mi2001 line (hereafter referred to as GFAP:GFP), was previously described^43^ and was obtained from the Zebrafish International Resource Center (ZIRC, Eugene, OR). The Tg(XlRho: EGFP) transgenic line (hereafter called XOPs:GFP) has been previously described^23^, and was obtained from James Fadool (Florida State University, Tallahassee, FL). The Tg(fli1a: EGFP)y1Tg transgenic line, (hereafter called fli1:GFP) has been previously described^44^, and was obtained from ZIRC. The Tg(atoh7:GFP) transgenic line, (hereafter called ath5:GFP) has been previously described^26^ and was obtained from ZIRC. Embryos were anesthetized with Ethyl 3-aminobenzoate methanesulfonate salt (MS-222, Tricaine, Sigma-Aldrich, St. Louis, MO) and adults were euthanized by rapid cooling as previously described^45^. All animal procedures and experimental protocols were approved and carried out in accordance with guidelines established by the University of Kentucky Institutional Animal Care and Use Committee (IACUC), the University of Kentucky Institutional Biosafety Committee, and the ARVO Statement for the Use of Animals in Ophthalmic and Vision Research.

### Generation of *her9* mutant zebrafish by CRISPR

The *her9* target sites and single strand DNA oligonucleotides used to generate the guide RNAs were selected using the ZiFit online tool (https://zifit.partners.org/ZiFiT/). The target sites for CRISPR/Cas9 genome editing were selected within the first and third exons of the *her9* gene. The first target site is 54 bp 3′ of the translation start site and 46 bp upstream of the beginning of the bHLH domain, while the second target site is immediately after the region corresponding to the Orange domain (sequences listed in Supplemental Table 2).

The pT3TS-nls-zCas9-nls (Addgene: 46757) expression vector was used to produce Cas9 mRNA. This vector contains a zebrafish codon-optimized Cas9 coding sequence flanked by the nuclear localization sequence. The plasmid was linearized using XbaI (NEB: R0145S) and purified by phenol:chloroform extraction and ethanol precipitation. Cas9 mRNA was generated using Ambion mMESSAGE mMACHINE T3 Transcription Kit (Life Technologies: AM1304) and purified by phenol:chloroform extraction and ethanol precipitation.

Screening for mutations in *her9* was performed by high resolution melting analysis (HRMA). Genomic DNA was collected from 24 hpf embryos as described below. HRMA was performed using the LightCycler 480 High-Resolution Melting Master (Roche) kit according to the manufacturer's instructions on a LightCycler 96 Real-Time PCR System (Roche).

### Genomic DNA (gDNA) extraction and amplification

gDNA was extracted from whole embryos or from tail clips of adult fish. The embryos or tails were placed in 20 µl of 1 × Thermopol buffer (NEB, Ipswich, MA) and incubated at 95°C to soften the tissue. The tissue was cooled on ice for 10 min before 5 µl of Proteinase K (Pro K; Sigma-Aldrich, St. Louis, MO) was added to each and incubated at 55°C overnight. After at least 18 h of digestion, tubes containing the digested tissue were incubated at 95º C for 15 min to deactivate the Pro K. The gDNA was then amplified using primers described in Supplemental Table 1.

### Restriction fragment length polymorphism (RFLP) analysis

The mutant *her9* allele carrying a 1 bp deletion was identified by RFLP analysis due to the introduction of a BsaJI restriction site (NEB: R0563S). The mutant *her9* allele carrying a 1 bp insertion was identified by RFLP due to the introduction of a BfaI restriction site (NEB: R0568S). Genomic DNA from whole embryos or from tail clips was extracted and amplified by PCR as described above, then subjected to restriction digest. The restriction digests were visualized on a 2% agarose gel.

### mRNA synthesis and microinjections

The coding region of *her9* was amplified by RT-PCR and the cDNA was cloned into a pGEMT-easy plasmid (Promega, Madison, WI). Capped mRNA was synthesized using the mMessage T7 kit (Ambion, Austin, TX) according to the manufacturer's instructions and purified by phenol–chloroform extraction and ethanol precipitation. The mRNA was injected at a volume of 4.18 nl/embryo (1.5 ng) in buffered solution with 0.025% Dextran red into the yolk of 1-cell stage zebrafish.

### Western blot analysis

Protein lysate was extracted from 20 heads of 3 dpf *her9* wildtype and mutant larvae. Protein was quantified by Bradford Assay (BIO-Rad), and 30 μg was diluted 1:3 in gel loading buffer (NEB), sonicated, spun down, then separated by SDS-PAGE on Mini-PROTEAN TGX Precast Gels (BIO-Rad). Following gel electrophoresis, separated proteins were transferred to a nitrocellulose membrane. The membrane was blocked with 5% non-fat milk/PBST for 30 min at room temperature then incubated in anti-human HES4 (rabbit polyclonal, 1:500, Thermo Fisher) or anti-β actin (mouse monoclonal, 1:1,000, Santa Cruz)/PBST solution overnight at 4 °C. Membranes were washed in PBST then incubated in secondary antibody for one hour at room temperature, developed with HRP Development Reagent (BIO-Rad) for 1 min, and imaged with a Chemidoc Bioimager (BIO-Rad). The experiment was performed in duplicate, and similar results were obtained both times.

### RNA isolation and riboprobe synthesis

RNA was isolated from whole embryos at indicated developmental time points. RNA was extracted from pools of embryos using TRIzol Reagent (Life Technologies, Invitrogen), following the manufacturer’s protocol, then treated with RNAse-Free DNAse I (Roche, Indianapolis, IN) to remove genomic DNA as previously described^46^. For riboprobes, PCR products from the unique regions of *her9* were cloned into the pGEMT-easy vector (Promega, Madison, WI). Plasmids were linearized using either NcoI and SpeI restriction enzymes (NEB, Ipswich, MA). Riboprobes were synthesized from the plasmids by in vitro transcription using either T7 or Sp6 polymerase and a digoxigenin (DIG) labeling kit (Roche Applied Science, Indianapolis, IN). The primer sequences used for riboprobe preparation are given in Supplemental Table 1.

### RT-PCR and real-time quantitative RT-PCR (qPCR)

The GoScript Reverse Transcriptase System (Promega, Madison, WI) was used to synthesize the first strand cDNA from 1 μg of the extracted RNA. PCR primers were designed to amplify unique regions of the *her9* and *atp5h* cDNAs (Eurofins Genomics; www.eurofinsgenomics.com; Supplemental Table 1). Faststart Essential DNA Green Master mix (Roche) was used to perform qPCR on a Lightcycler 96 Real-Time PCR System (Roche). The relative transcript abundance was normalized to *atp5h* expression as the housekeeping gene control^47^, and was calculated as fold-change relative to 36 h post fertilization (hpf) for developmental expression, and fold-change relative to wild type siblings, untreated, or uninjected embryos for the mRNA rescue and RA experiments. RT-PCR and qPCR experiments were performed with three biological replicates and three technical replicates. RT-PCR was performed on a Mastercycler Pro thermocycler (Eppendorf, Westbury, NY). PCR products were visualized on a 1% agarose gel.

### Visually mediated background adaptation assay (VBA assay)

The VBA assay was adapted from previously described protocols^19,20^. Embryos at 5 dpf were incubated in the dark for 2 h and the dorsal cranial pigment was immediately imaged after removal from the dark. The embryos were then placed under ambient light for 30 min. The dorsal cranial pigment was imaged immediately following light exposure. Images of the heads of the embryos were scored by naïve observers as either "Light" or "Dark". All imaged embryos were then genotyped by RFLP analysis. The assay was performed a minimum of three times. In each assay, 10 embryos were screened. Results were analyzed by χ^2^ test.

### Optokinetic response assay (OKR assay)

Embryos were collected at 5 dpf and mounted in a 35-mm petri dish filled with 5% methylcellulose. The embryos were mounted near the surface of the methylcellulose, dorsal side up. The petri dish was then placed inside of a rotating drum containing illuminated vertical stripes. The drum was rotated clockwise for 30 seconds at 8 rpm then 30 seconds counterclockwise at 8 rpm and finally clockwise for another 30 seconds. Embryos were scored "responders" if they displayed smooth saccade eye movements in response to the drum rotation in the direction of rotation for at least two of the 30 second intervals. Embryos were scored as “non-responders” if they displayed no eye movement in any of the 30 second intervals. After scoring, the embryos were collected in 1 × Thermopol buffer for gDNA extraction and genotyping using RFLP analysis as described above. The OKR assay was performed a minimum of three times, and at least 10 embryos were scored each time.

### Mobility assay

Single 5 dpf zebrafish larvae were filmed for 1 min in a 96-well plate to assess their swimming behavior. Each larva was subsequently screened for the *her9* 1 bp insertion via PCR amplification and a restriction digest with BfaI (NEB: R0568S). The videos were linked to the appropriate genotype and analyzed using EthoVision software (www.noldus.com/animal-behavior-research/products/ethovision-xt). EthoVision analysis produced individual tracks for each larva, a compiled heat map of movement by genotype, and comparisons of total velocity and distance traveled.

### Whole-mount and fluorescent in situ hybridization (WISH and FISH)

Embryos were manually dechorionated, collected at selected developmental time points (18, 24, 48, 72, and 120 hpf) and fixed as described above. WISH was performed as previously described^48^. Solutions were prepared with diethyl pyrocarbonate (DEPC)-treated water. DIG-labeled riboprobes (3 ng/μl) were hybridized to the samples overnight at 60 °C in hybridization buffer. After washing and blocking, samples were incubated overnight at 4 °C with an anti-DIG-AP antibody (Roche, diluted 1:2,000 in blocking solution). The next day, the embryos were washed and equilibrated in NTMT buffer followed by coloration with 4-nitro blue tetrazolium (NBT, Roche) and 5-Bromo-4chloro-3-indolyl-phosphate, 4-toluidine salt (BCIP, Roche) in NTMT buffer. A stop solution (PBS pH 5.5, 1 mM EDTA) was used to end the coloration reaction and embryos were placed in 40% glycerol for imaging.

FISH was performed as previously described^49^. Embryos were fixed and sectioned as previously described^48^. Sections were post-fixed in 1% PFA and rehydrated in PBST. The TSA plus Cy3 Kit (Perkin Elmer Inc, Waltham, MA) was used for probe detection. The sections were counterstained with 4′, 6-diamidino-2-phenylindole (DAPI, 1:10,000 dilution, Sigma-Aldrich), mounted in 60% glycerol, and imaged on an inverted fluorescent microscope (Nikon Eclipse Ti-U, Nikon Instruments, Melville, NY) using a 20 × or 40 × objective and a Leica SP8 DLS confocal/digital light sheet system (Leica Biosystems, Nussloch, Germany) using a 40 × or 63 × objective. At least 10 embryos and 10 sections were analyzed for each time point and probe.

### Immunohistochemistry

Immunohistochemistry was performed as previously described^50^. The following primary antibodies were used: 4C12 (mouse, 1:100) generously provided by J. Fadool (Florida State University, Tallahassee, FL), which labels rod photoreceptor cell bodies; 1D1 (mouse, 1:100, J. Fadool, FSU, Tallahassee, FL), which recognizes rhodopsin; Zpr-1 (mouse, 1:20, ZIRC), which labels red-green double cones; HuC/D (mouse, 1:20, Invitrogen, Grand Island, NY), which recognizes retinal ganglion cells and amacrine cells; Prox1 (rabbit, 1:2,000, Millipore, Billerica, MA), which recognizes horizontal cells; anti-PCNA (mouse, 1:100, Santa Cruz Biotechnology, Dallas, TX), which labels proliferating cells; Zpr-3 (rabbit, 1:1,000, ZIRC), which labels double cone and rod photoreceptor outer segments; and anti-Blue, -green, and -UV Opsin (rabbit, 1:1,000), generously provided by D. Hyde (University of Notre Dame) which labels the blue, green, and UV cones, respectively. Alexa Fluor 488 goat anti-mouse, 488 goat anti-rabbit, 546 goat anti-rabbit, and 546 goat anti-mouse secondary antibodies (Molecular Probes, Invitrogen) were all used at 1:200 dilution. Nuclei were visualized by counterstaining with DAPI (1:10,000 dilution). Samples were mounted in 60% glycerol in PBS. Images were taken at 20 × and 40 × on an inverted fluorescent microscope and a Leica SP8 DLS confocal/digital light sheet system using a 40 × or 63 × objective. Ten sections were analyzed on each slide and for each antibody.

### TUNEL staining

Terminal deoxynucleotide transferase (TdT)-mediated dUTP nick end labeling (TUNEL) was performed on frozen retinal cryosections using the ApopTag Fluorescein Direct In Situ Apoptosis Detection Kit (Millipore, Billerica, MA). TUNEL staining was performed according to the manufacturer's protocol. Images were taken at 20 × and 40 × on an inverted fluorescent microscope (Eclipse Ti-U, Nikon instruments). Ten sections were analyzed on each slide.

### RA treatment

Stocks of all-trans-RA in dimethyl-sulfoxide (DMSO) were thawed before use and diluted to 1 µM retinoic acid with 0.3% DMSO in embryo medium, 100 µM of the RA signaling inhibitor diethylaminobenzaldehyde (DEAB) in 0.3% DMSO in embryo medium or 0.3% DMSO alone as the carrier control. Fli1:EGFP embryos and embryos from *her9* heterozygous incrosses were immersed in the RA solution starting at 24 hpf and were incubated for 12 h in complete darkness. At 36 hpf the embryos were rinsed in artificial fish water for 10 min and processed for immunohistochemistry, or the heads were removed and processed for qPCR.

To observe effects of RA on rod and cone photoreceptors, embryos from wild type or *her9* heterozygous incrosses were incubated in 0.3 μM RA solution starting at 48 hpf and the solution was refreshed every 24 hpf. The embryos were collected at 72 hpf for analysis of rods, and at 120 hpf for analysis of cones. The heads were removed and sectioned for IHC and imaging.

### Cell counts

For comparing and counting retinal cells, only retinal cryosections containing the optic nerve as a landmark were used for cell counts. For photoreceptor and horizontal cells, counts were taken from a 100 µm region of the outer retina, beginning 100 μm dorsal to the optic nerve. For Müller glia, ganglion and amacrine cells, counts were taken from a 100 µm region directly adjacent to the optic nerve. For each experiment, sections were counted from a minimum of 10 wild-type, heterozygous or mutant embryos.

### Statistical analysis

Statistical analysis was performed using a Student’s t-test or χ^2^ test with GraphPad Prism software (www.graphpad.com). For comparing the number of retinal cell types and other phenotypic features 10 wild type and 10 mutant animals were examined. Boxplot graphs were prepared using ggplot2 in R (version 3.6.2; https://www.R-project.org/). All graph data are represented as the mean ± the standard deviation (s.d.).

## Supplementary information

Supplementary information
