# Author Correction: Her9/Hes4 is required for retinal photoreceptor development, maintenance, and survival

**DOI:** 10.1038/s41598-020-72314-x

**Published:** 2020-09-04

**Authors:** Cagney E. Coomer, Stephen G. Wilson, Kayla F. Titialii-Torres, Jessica D. Bills, Laura A. Krueger, Rebecca A. Petersen, Evelyn M. Turnbaugh, Eden L. Janesch, Ann C. Morris

**Affiliations:** Department of Biology, University of Kentucky, 215 T.H. Morgan Building, Lexington, KY 40506-0225 USA

Correction to: *Scientific Reports* 10.1038/s41598-020-68172-2, published online 09 July 2020

The Supplementary Information file that accompanies this Article contains errors in Supplemental Table 1.

The primer sequence reported for *her9* F, “CCTGACGGAGAACTGAACACAAGACACACA”

should read:

“CGCCACACACACGCTCGTGT”

The sequence reported for *her9* R, “TTTCTCAATGGTACGGCGGGTGCTCTGGGC”

should read:

“CGGCTTGTGGAACGCCCGAA”

The sequence reported for *her9* CR F, “AAGCTTCCTGACGGAGAACTGAACACAAGACACACA”

should read:

“AAGCTTTGGCTTCTACCGGACTCAACTTTGGTGTTT”

Finally, the sequence reported for *her9* CR R, “GAATTCTTTCTCAATGGTACGGCGGGTGCTCTGGGC”

should read:

“GAATTCCCATGAAAACTTTATAAGTTCATATGAGATGCGC”

