# Supplementary material for: Her9/Hes4 is required for retinal photoreceptor development, maintenance, and survival
Source: Sci Rep. 2020 Jul 9;10:11316. doi: 10.1038/s41598-020-68172-2 (PMC7347560; doi:10.1038/s41598-020-68172-2)
Supplement: Supplementary file 1 — Supplementary information [file 41598_2020_68172_MOESM1_ESM.docx]

**Her9/Hes4 is required for retinal photoreceptor development, maintenance, and survival**

Cagney E. Coomer, Stephen G. Wilson, Kayla F. Titialii-Torres, Jessica D. Bills, Laura A. Krueger, Rebecca A. Petersen, Evelyn M. Turnbaugh, Eden L. Janesch and Ann C. Morris

Department of Biology, University of Kentucky, Lexington, Kentucky 40506

**Supplementary Information**

**Supplemental Figures and Legends**

**
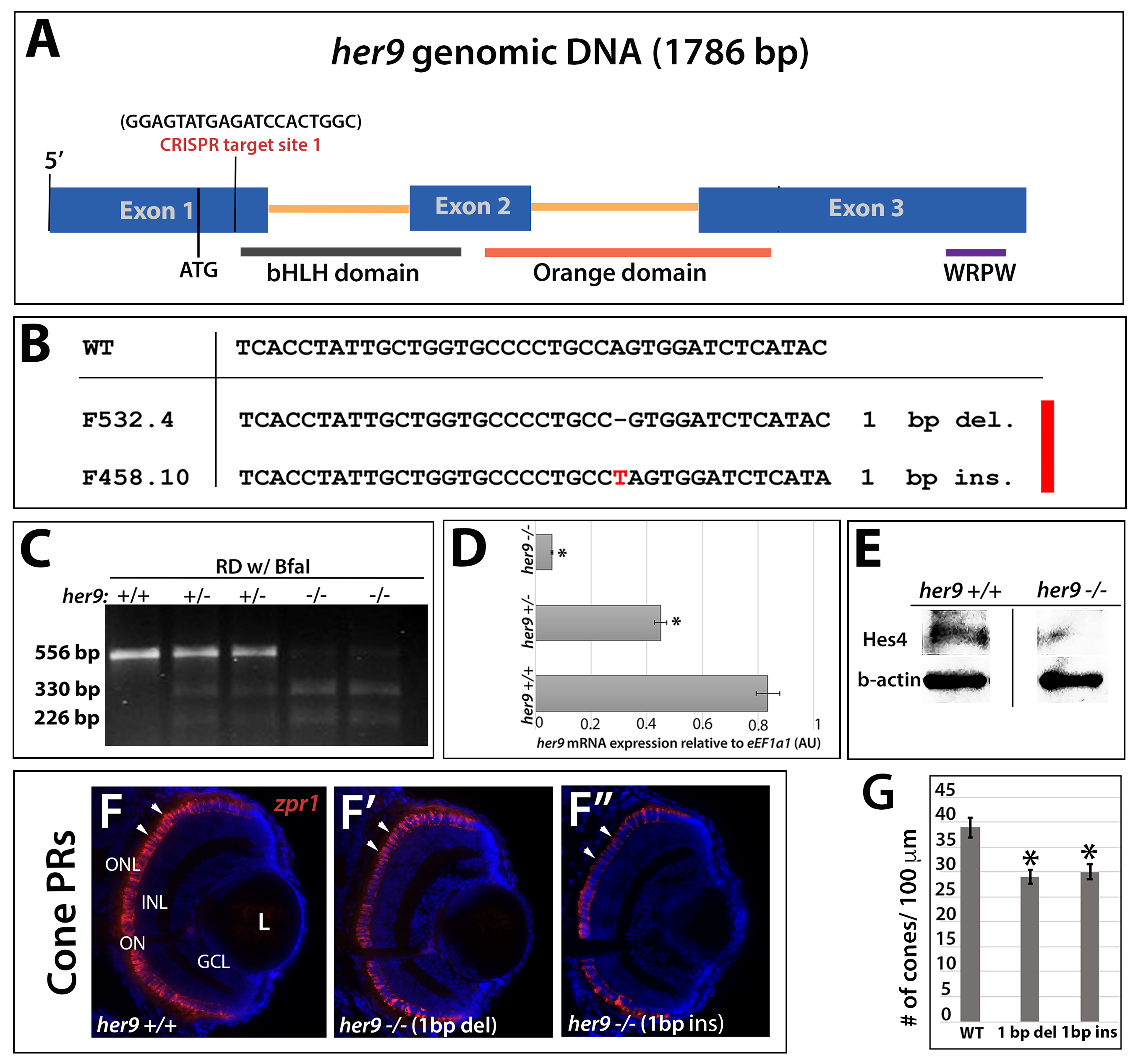
**

**Figure S1. Generation of *her9* mutants using CRISPR/Cas9. (A)** The *her9* locus contains 3 exons spanning 1786 bp on linkage group 23. The first CRISPR target site is 54 bp 3’ of the translation start site, and 46 bp upstream of the beginning of the bHLH domain. **(B)** Comparison of WT *her9* sequence with 1bp deletion and 1bp insertion mutations. (**C)** RFLP analysis of WT, heterozygous and homozygous *her9* 1 bp insertion mutant cut with BfaI. (**D)** qPCR analysis of *her9* mRNA expression in WT, heterozygous and homozygous mutants at 48 hpf. (**E)** Western blot with a HES4 antibody, indicating the loss of Her9 protein in the *her9* mutants compared to WT siblings. Immunohistochemistry with a red-green cone antibody (Zpr1) in *her9*^+/+ or +/-^ and *her9*^-/-^ (1 bp ins and 1 bp del; **F-F’)** retinal sections. **(G)** Cone cell counts in *her9^-/-^* mutant larvae with 1 bp ins and 1 bp del and their WT siblings ( # of cones/ 100 µm; t-test (p< .0001).

**
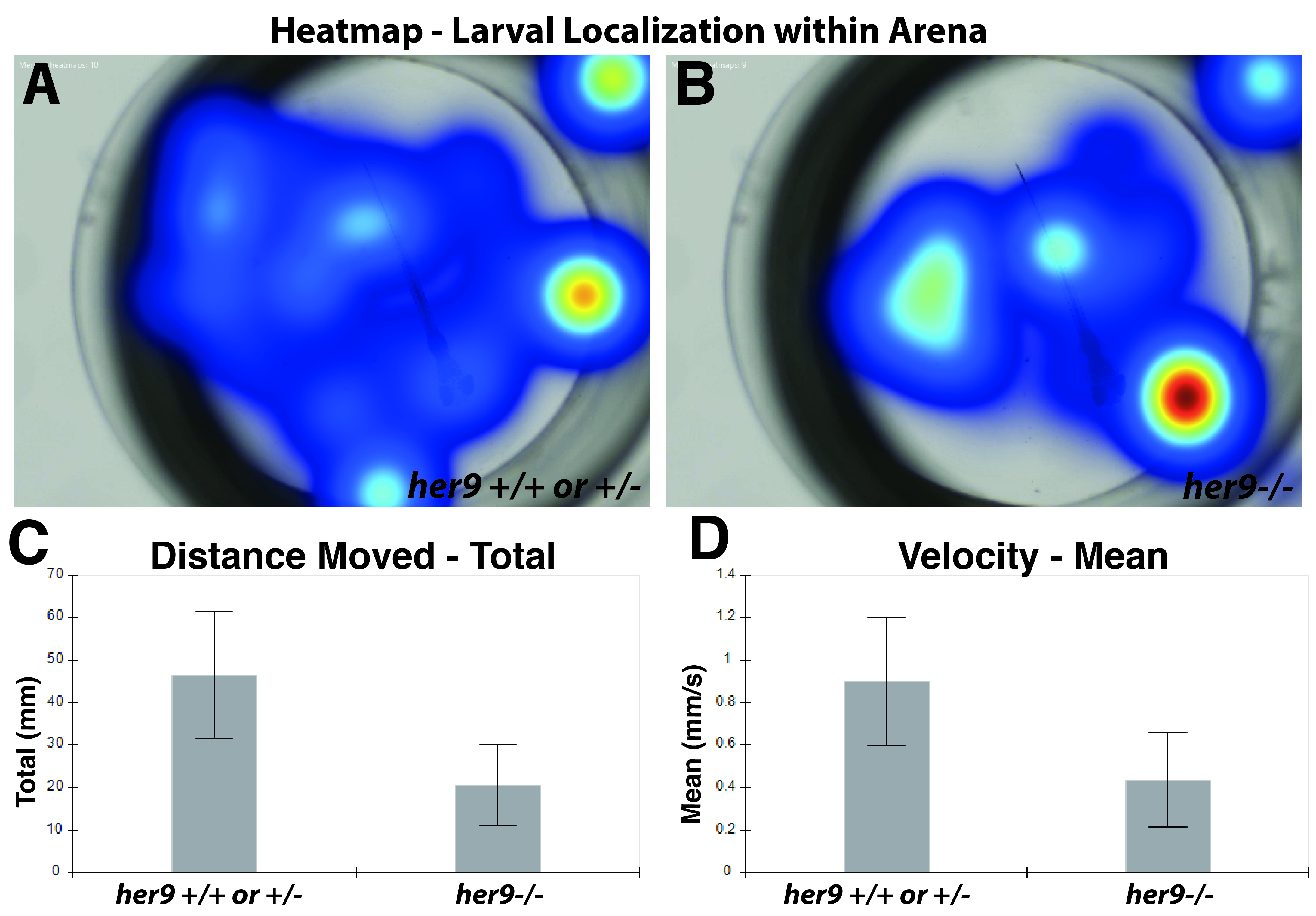
**

**Figure S2. Mobility assay of 5 dpf larvae. (A)** Heat map indicating the amount of time the WT or *her9*+/- larva spends in different parts of the arena. **(B)** Heat map indicating the amount of time the *her9*-/- larva spends in different parts of the arena. **(C)** Comparison of the average total distance travel by the larvae. The WT or *her9* +/- average total distance traveled was 46.47±14.91 mm, the *her9* -/- average total distance traveled was 20.59±9.59 mm. WT/Het= 10 embryos; Mut = 9; t-test (p=0.0824). **(D)** Comparison of the average velocity of larvae. The WT or *her9*+/- average velocity was 0.901±0.302mm/s, the *her9*-/- average velocity was 0.436±0.22 mm/s. WT/Het= 10 embryos; Mut = 9 embryos; t-test (p= 0.1162).

**
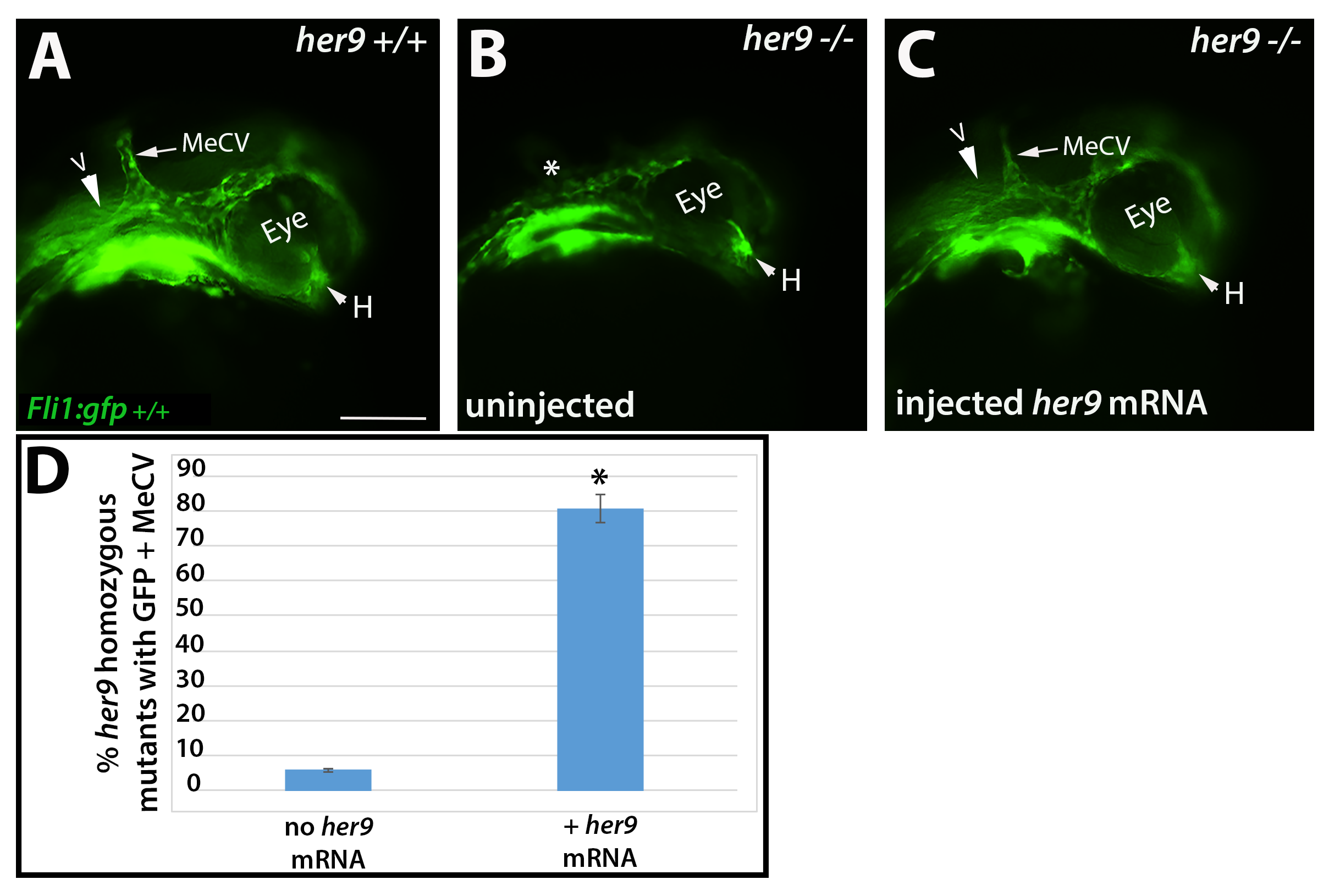
**

**Figure S3. Injection of *her9* mRNA rescues the *her9* mutant phenotype. (A)** Fli1:GFP+ midcerebral vein in WT embryos at 24 hpf. (**B)** Missing midcerebral vein in uninjected *her9* homozygous mutant embryos at 24 hpf (asterisk; n=193 embryos; WT=48, Het=101, Mut= 44; MeCV+=160; MeCV-=33; **χ^2^** = p< .00001). (**C)** In *her9* homozygous mutants injected with *her9* mRNA, the midcerebral vein is now visible at 24 hpf. (**D)** Quantification of rescue of *her9* mutant phenotype after *her9* mRNA injections (n=47embryos; MeCV+=42; **χ^2^** = p< 0.000508). MeCV, Midcerebral vein; H, hyaloid vein; V, ventricle. Scale bar= 50 µm.

**
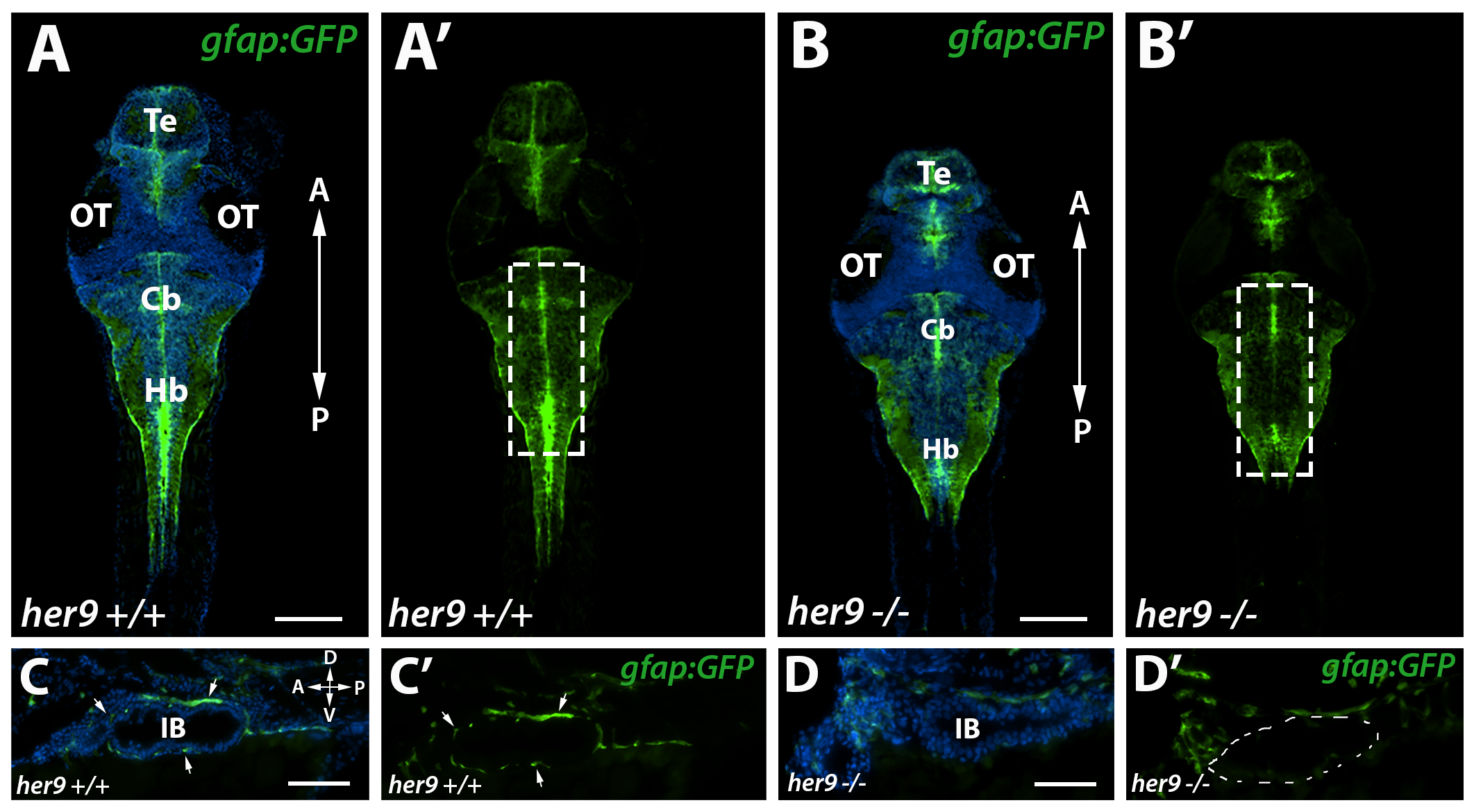
**

**Figure S4. Her9 mutants display decrease in glial cells in brain and gut. (A-B’)** Cryo-sections of *her9^-/-^* mutant larvae and their WT siblings brain on *gfap:GFP* background (dash lined box highlights *gfap* expressing cells in Cb and Hb). **(C-D’)** Cryo-sections of *her9^-/-^* mutant larvae and their WT siblings brain on *gfap:GFP* background (Arrows indicate gfap expressing cells in Intestinal Bulb). Te, Telencephalon; OT, Optic tectum; Cb, Cerebellum; Hb, Hindbrain; IB, Intestinal Bulb. Scale bar= 50 µm

**
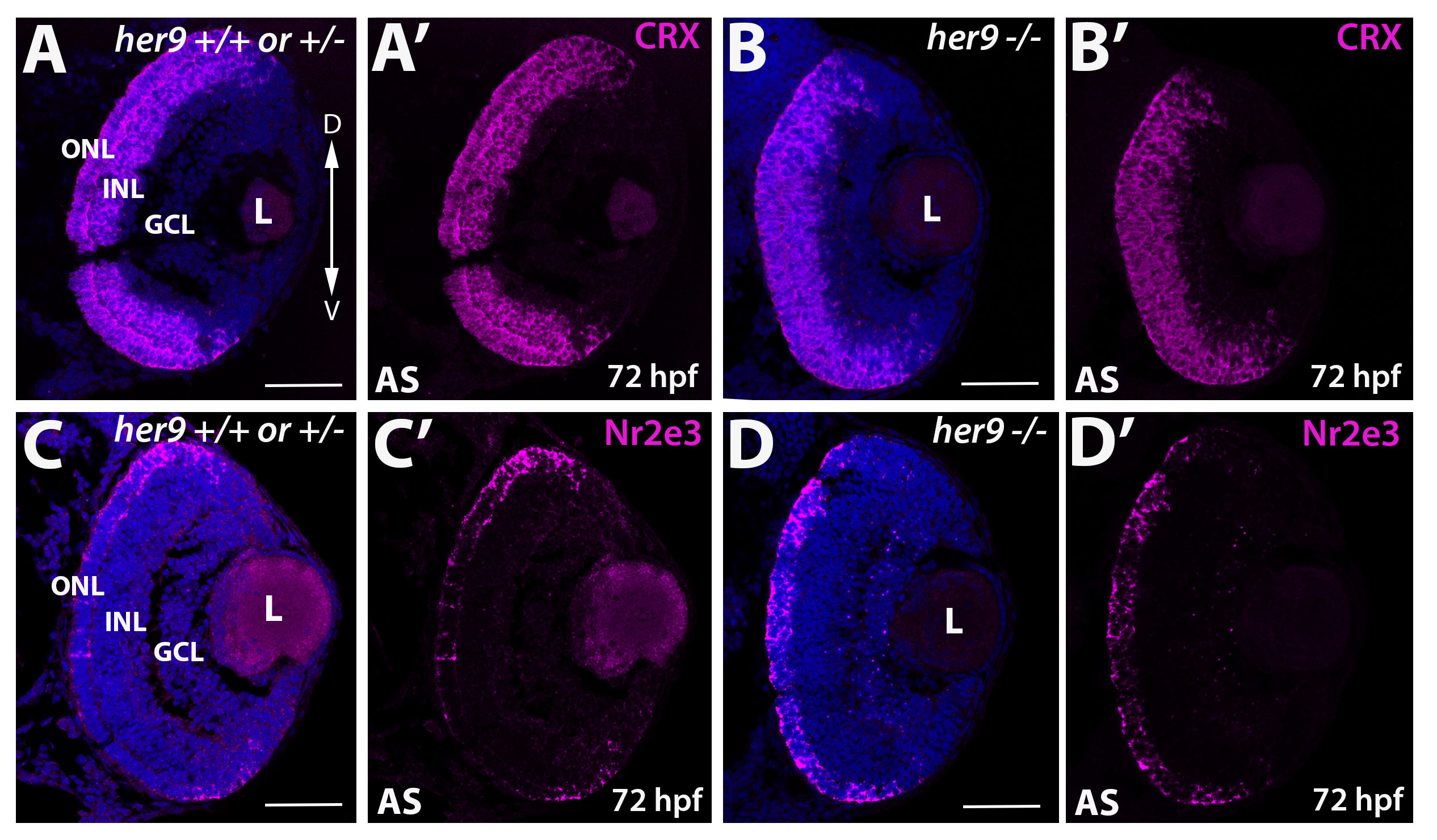
**

**Figure S5. Expression of *crx* and *Nr2e3* at 72 hpf.** (**A-B)** Fluorescent in situ hybridization (FISH). *Crx* expression at 72 hpf in WT and *her9* mutant retina. *Her9* mutants displayed similar expression to WT. **(C-D’)** *Nr2e3* expression at 72 hpf in WT and *her9* mutant retina. Expression in the mutant ONL was distorted compared to the WT. L, lens; ON, optic nerve. Scale bar= 50 µm

**
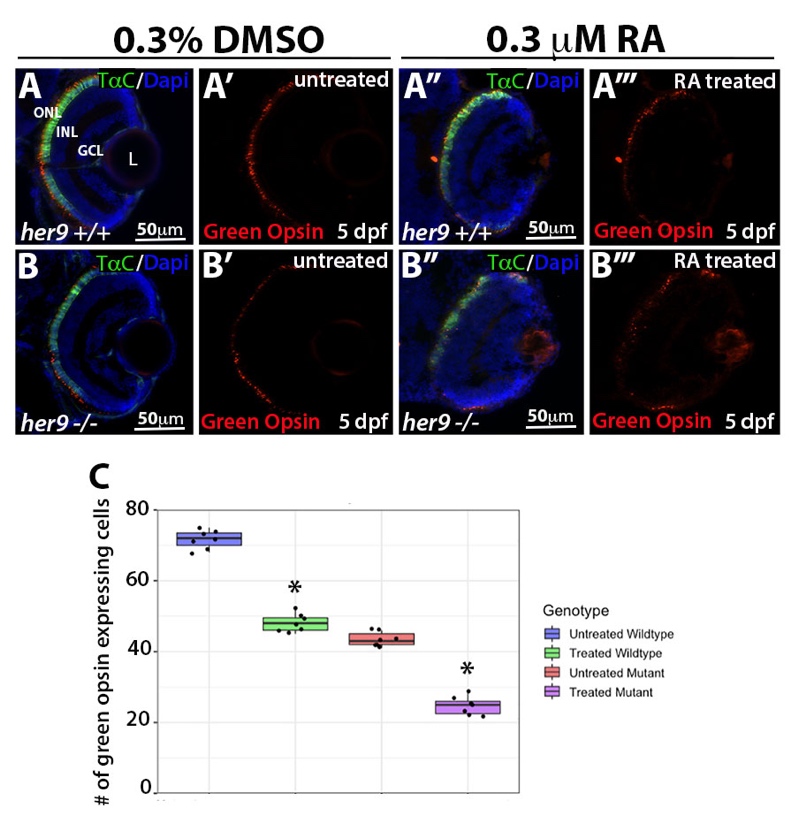
**

**Figure S6. Her9 is not required for the effects of RA on opsin expression. (A-A’’’)** IHC on TαC:GFP WT retinas comparing Green opsin expression after being treated with RA from 24- 120 hpf. **(B-B’’’)** IHC on TαC:GFP mutant retinas comparing Green opsin expression after being treated with RA from 24- 120 hpf. **(C)** Cell counts comparing Green opsin expressing cells in the untreated and treated retinas. WT/Het (untreated) = 10; WT/Het (treated) = 10; t-test (p< .0001). Mut (untreated) = 10; Mut (treated) = 10; t-test, *p< .0001. ONL, outer nuclear layer; INL, inner nuclear layer; GCL, ganglion cell layer; L, lens; Scale bar= 50µm


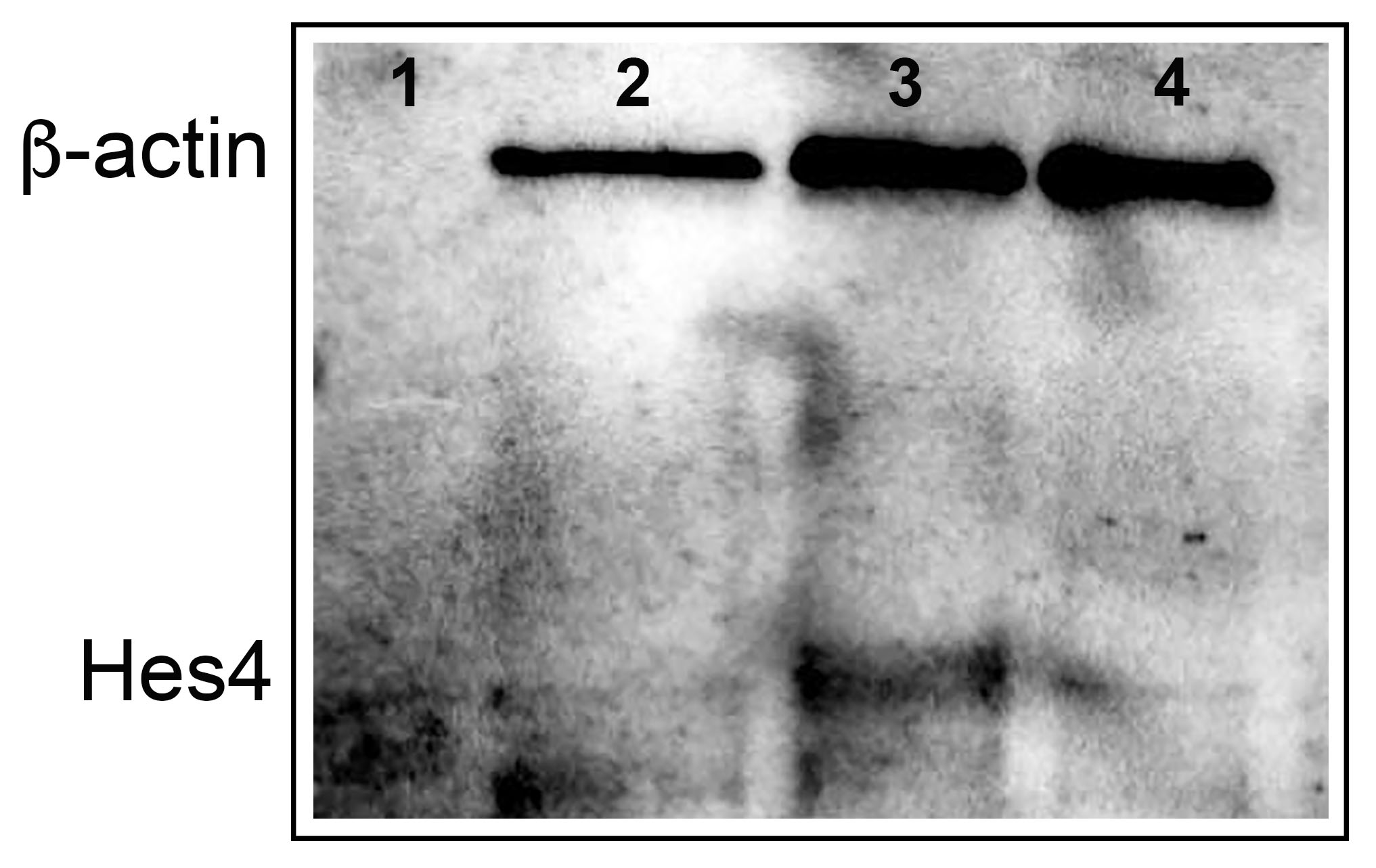


**Figure S7. Western blot. (1)** Ladder. **(2)** Adult zebrafish Pancreatic lysate (Positive control). **(3)** Her9 +/+ or +/- Lysate (Upper band b-actin; lower band Hes4). **(4)** Her9 -/- Lysate (Upper band b-actin; lower band Hes4). Small band in Mutant lane could be non-specific.


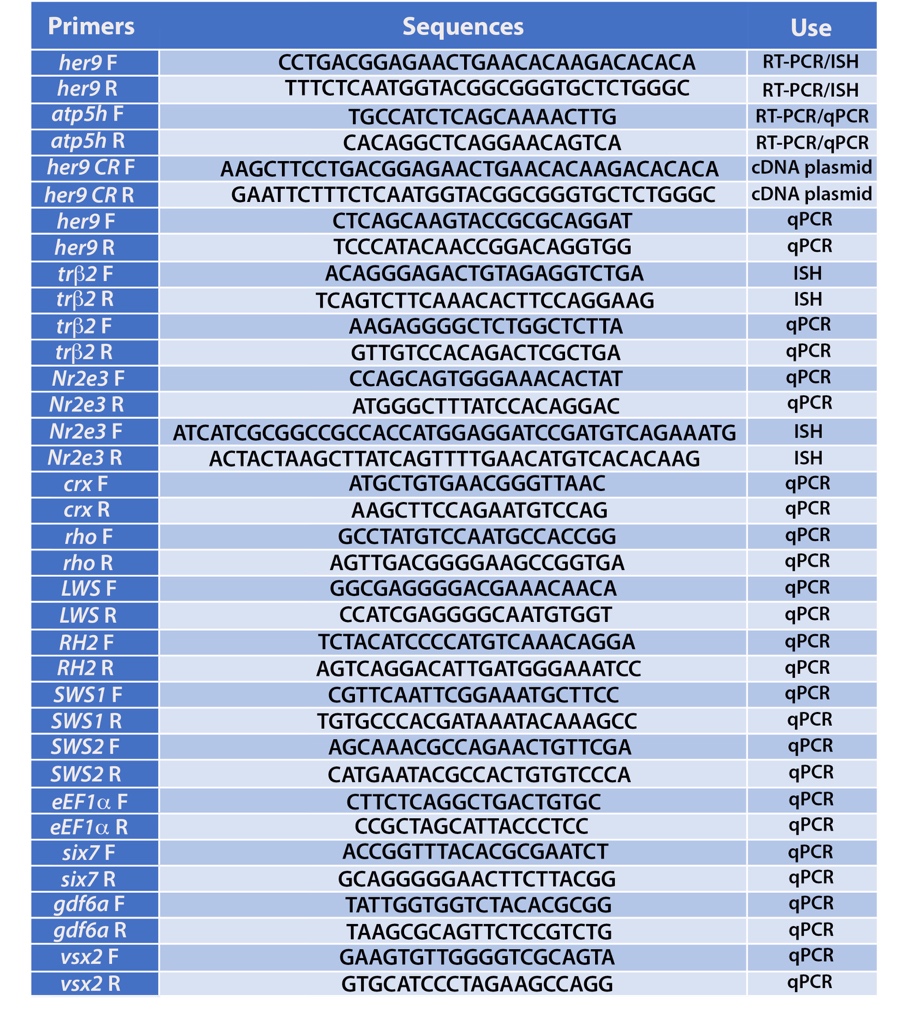


**Supplemental Table 1.** Primer sequences used for RT-PCR and qPCR. RT-PCR primers were also used to design WISH and FISH probes. F, forward; R, reverse; CR, coding region.

**
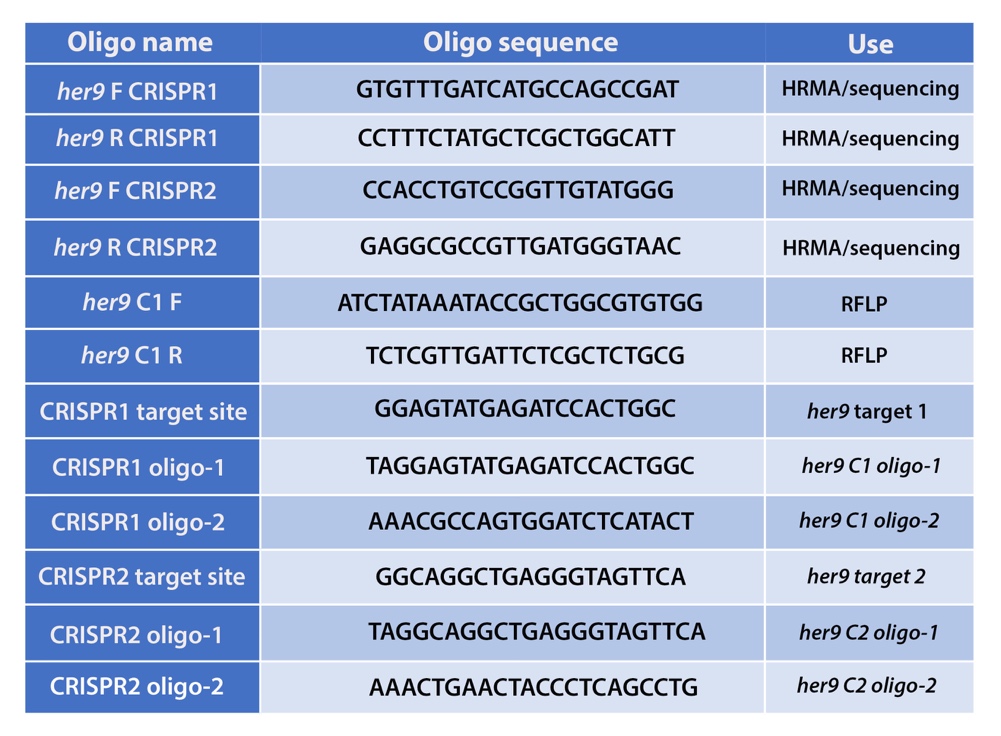
**

**Supplemental Table 2.** Oligo sequences used for her9 CRIPSR. F, forward; R, reverse; C1, CRISPR1.
